# Neonatal *Scn1b-*null mice have sinoatrial node dysfunction, altered atrial structure, and atrial fibrillation

**DOI:** 10.1172/jci.insight.152050

**Published:** 2022-05-23

**Authors:** Roberto Ramos-Mondragon, Nnamdi Edokobi, Samantha L. Hodges, Shuyun Wang, Alexandra A. Bouza, Chandrika Canugovi, Caroline Scheuing, Lena Juratli, William R. Abel, Sami F. Noujaim, Nageswara R. Madamanchi, Marschall S. Runge, Luis F. Lopez-Santiago, Lori L. Isom

**Affiliations:** 1 Department of Pharmacology and; 2 Department of Internal Medicine, Division of Cardiovascular Medicine, University of Michigan Medical School, Ann Arbor, Michigan, USA.; 3 Department of Molecular Pharmacology & Physiology, University of South Florida College of Medicine, Tampa, Florida, USA.; 4 Department of Neurology and; 5 Department of Molecular & Integrative Physiology, University of Michigan Medical School, Ann Arbor, Michigan, USA.

**Keywords:** Cardiology, Arrhythmias, Mouse models, Sodium channels

## Abstract

Loss-of-function (LOF) variants in *SCN1B*, encoding the voltage-gated sodium channel β1/β1B subunits, are linked to neurological and cardiovascular diseases. *Scn1b*-null mice have spontaneous seizures and ventricular arrhythmias and die by approximately 21 days after birth. β1/β1B Subunits play critical roles in regulating the excitability of ventricular cardiomyocytes and maintaining ventricular rhythmicity. However, whether they also regulate atrial excitability is unknown. We used neonatal *Scn1b-*null mice to model the effects of *SCN1B* LOF on atrial physiology in pediatric patients. *Scn1b* deletion resulted in altered expression of genes associated with atrial dysfunction. *Scn1b*-null hearts had a significant accumulation of atrial collagen, increased susceptibility to pacing induced atrial fibrillation (AF), sinoatrial node (SAN) dysfunction, and increased numbers of cholinergic neurons in ganglia that innervate the SAN. Atropine reduced the incidence of AF in null animals. Action potential duration was prolonged in null atrial myocytes, with increased late sodium current density and reduced L-type calcium current density. *Scn1b* LOF results in altered atrial structure and AF, demonstrating the critical role played by *Scn1b* in atrial physiology during early postnatal mouse development. Our results suggest that *SCN1B* LOF variants may significantly impact the developing pediatric heart.

## Graphical abstract

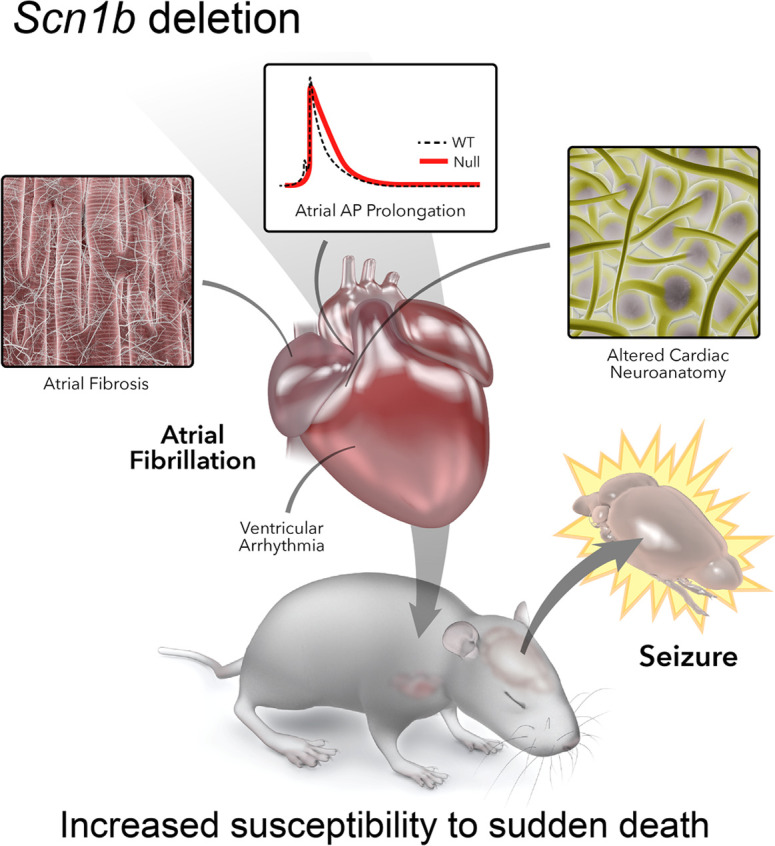

## Introduction

Loss-of-function (LOF) variants in *SCN1B* (19q13.11), encoding the voltage-gated sodium channel (VGSC) β1/β1B subunits, are linked to human diseases that predispose patients to sudden death, including developmental and epileptic encephalopathy type 52 (DEE52; OMIM 617350), Genetic Epilepsy with Febrile Seizures plus (OMIM 617350), Brugada Syndrome 5 (OMIM 612838), cardiac conduction defects (OMIM 612838), and Atrial Fibrillation (AF) Familial 13 (OMIM 615377) (1, 2). *Scn1b*-null mice phenocopy DEE52, which is associated with biallelic inheritance of *SCN1B* LOF variants (3–5), with severe seizures developing 10 days after birth and 100% mortality in the third week of life (6). Consistent with evidence linking *SCN1B* variants to human cardiac disease, *Scn1b*-null mice also have cardiac arrhythmias that include bradycardia and prolonged Q-T intervals (7). Acutely isolated neonatal *Scn1b*-null ventricular cardiomyocytes have increased transient and late sodium current (*I_Na,T_* and *I_Na,L_*) densities, reduced calcium current (*I_Ca_*) density, an altered potassium current (*I_K_*) profile, and altered calcium handling, resulting in the generation of arrhythmogenic substrates (7–9). These results, together with our work in other models of sodium channelopathies (10, 11), led us to propose that cardiac arrhythmias contribute to the mechanism of Sudden Unexpected Death in Epilepsy (SUDEP) in patients with DEE with VGSC gene variants.

Non-pore-forming VGSC β subunits are multifunctional (12). They are transmembrane proteins with type 1 topology consisting of an extracellular N-terminus containing an Ig domain, a single transmembrane segment, and a C-terminal intracellular domain (ICD) (13). *SCN1B* encodes β1 as well as the secreted splice variant β1B, which includes a retained intron (14, 15). β1 subunits associate with multiple VGSC α subunits to regulate their cell-surface expression and modulate *I_Na_* (13). β1B subunits appear to be more selective; they regulate Na_v_1.3- and Na_v_1.5-generated *I_Na_* in heterologous systems (15, 16). β1 Subunits also associate with potassium channels to regulate *I*_K_ (9, 17, 18). While β1 association with calcium channels has not been investigated, *Scn1b*-null ventricular myocytes have reduced *I_Ca_* (9). β1 subunits also play nonconducting roles as Ig superfamily cell adhesion molecules, participating in cell-cell and cell-matrix adhesion to regulate cardiac intercalated disk formation as well as neuronal pathfinding and fasciculation in the brain (19, 20). β1B functions as a secreted cell adhesion molecule ligand that has developmentally regulated expression patterns (15). Finally, β1 subunits undergo regulated intramembrane proteolysis (RIP) through sequential cleavage by the β-site Amyloid Precursor Protein (APP) cleaving enzyme-1 (BACE1) and γ-secretase (9, 21). These cleavage events release the β1 extracellular Ig domain, which functions as a ligand for cell adhesion (15, 22) as well as a soluble ICD that translocates to the nucleus to participate in transcriptional regulation of a group of genes, including those encoding ion channels, in the cardiac ventricle (9).

Our previous work focused on the role of *Scn1b* in neurons and cardiac ventricles. Here, we explored whether *Scn1b* also regulates cardiac atrial excitability to understand its potential role in the development of AF, the most common type of cardiac arrhythmia and a significant contributor to mortality (23, 24). Our results suggest that *Scn1b*-null neonatal atria have altered excitability, with changes in *I_Na,L_*, L-type *I*_Ca_, (*I_Ca,L_*), fibrosis, and cardiac innervation, which all contribute to the early development of AF. Our work introduces a potentially new role for *SCN1B* in the regulation of atrial rhythmicity and adds to the growing body of literature suggesting that LOF variants in *SCN1B* predispose patients to a compromised cardiovascular system, in addition to epilepsy, which, combined, may contribute to the mechanism of SUDEP.

## Results

### Differentially expressed genes in Scn1b-null versus WT atria.

**Figure 1:**
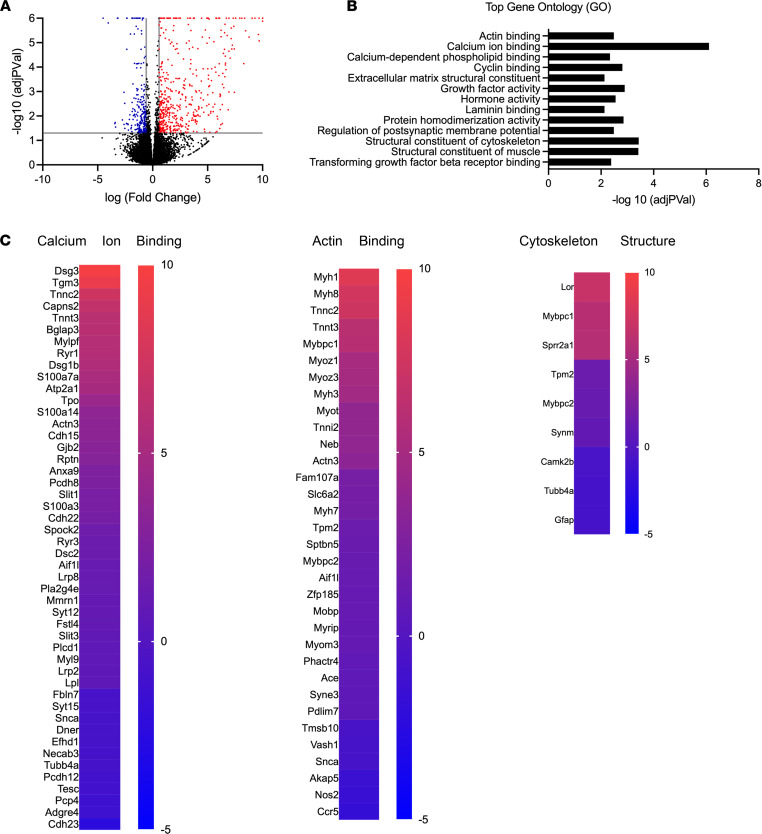
Differential gene expression in P16 *Scn1b*-null versus WT atria. (**A**) Volcano plot showing upregulated (red) and downregulated (blue) gene expression in *Scn1b*-null atrium compared with WT. (**B**) GO groups overrepresented in analysis of *Scn1b*-null atrium compared with WT. (**C**) Heat maps depicting altered genes related to calcium ion binding, actin binding, and cytoskeleton structure.

In the human heart, *SCN1B* mRNA expression is 3 times higher in atria than in ventricles (25), suggesting that *SCN1B* variants may result in atrial as well as ventricular arrhythmias. We repeated these expression experiments in a P16 WT mouse heart and found similar results (Supplemental Figure 1; supplemental material available online with this article; https://doi.org/10.1172/jci.insight.152050DS1). In previous work, we showed that VGSC β1 subunits are sequentially cleaved by BACE1 and γ-secretase and that the cleaved β1 intracellular domain translocates to the nucleus to contribute to transcriptional regulation of multiple gene families, including ion channels, in the mouse cardiac ventricle (9). Here, we used paired-end RNA-Seq to determine the differential gene expression profiles of *Scn1b*-null versus WT mouse atrial tissue (Figure 1). We performed these experiments at P16, as *Scn1b*-null mice die before the end of the third week after birth. The data were normalized, and differential expression analysis was performed with DESeq2 as a fee-for-service by the University of Michigan Advanced Genomics Core. A total of 724 genes were found to be differentially expressed between null and WT atrial tissues (Figure 1A). Gene Ontology (GO) analysis revealed groups of genes with altered expression in response to *Scn1b* deletion (Figure 1B). The sets of genes most differentially expressed between genotypes included those involved in calcium ion binding, actin binding, and cytoskeleton structure (Figure 1C). A number of these genes have been implicated in cardiac disease. Some of the most upregulated individual genes, *Sprr1a* and *Sprr2a1-3,* each with a LogFC greater than 5.5*,* belong to the family of small proline-rich proteins (SPRR). Upregulation of *Sprr* expression has been linked to the progression of fibrosis in pathological hypertrophy (26). In addition, we found several upregulated genes that are implicated in cardiac disease, including bone morphogenetic protein 4 (*Bmp4*) (27) (LogFC 0.728), α-actin (*Acta1*) (LogFC 3.335), and myosin heavy chain β (*Myh7*) (28–30) (LogFC 1.954). We confirmed the differential expression of a subset of genes at P16 between genotypes using quantitative PCR (qPCR) (Supplemental Figure 2). We also found significant differences in the expression of genes encoding select potassium (*Kcnu1*, *P* = 0.006; *Kcnd*2, *P* = 0.039; *Kcnt1*, *P* = 0.001; and *Kcnv1*, *P* = 0.045) and calcium channel (*Cacng1*, *P* = 1 × 10^–6^) subunits (Supplemental Figure 3). Taken together, these data suggest that neonatal *Scn1b*-null atria may have structural and electrical abnormalities that generate substrates for arrhythmia.

### Altered atrial structure in the Scn1b-null mice.

**Figure 2:**
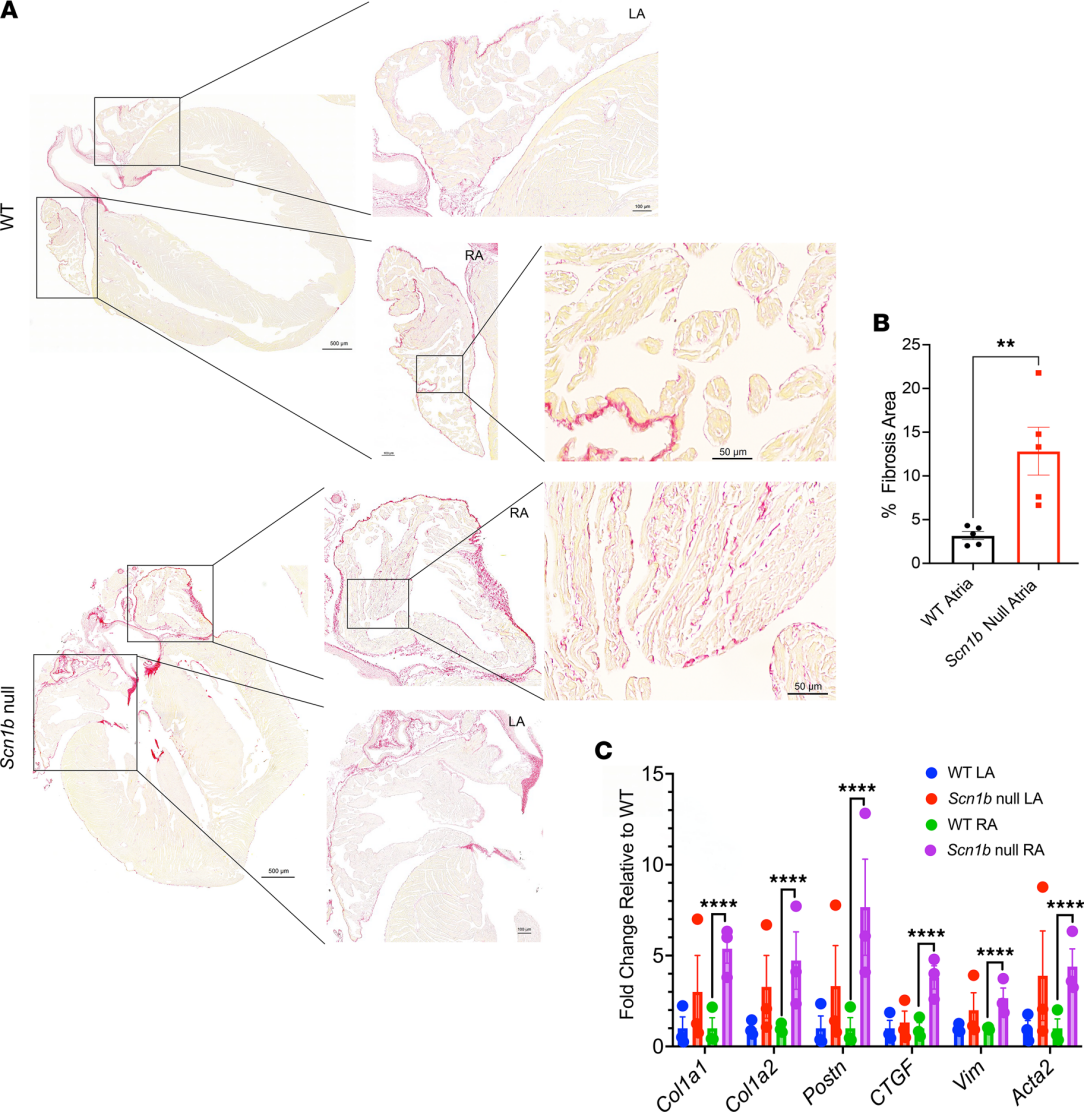
Fibrosis in P16–17 WT and *Scn1b*-null mouse hearts. (**A**) Representative images of histological coronal sections of whole heart fibrosis with picrosirius red staining. Insets show left and right atria. (**B**) Percent fibrosis area in WT and *Scn1*b-null mouse atria (*n* = 5). (**C**) Transcript levels of *Col1a1*, *Col1a2*, *Postn*, *CTGF*, *Vim*, and *Acta2* in the left versus right atria from WT and *Scn1b*-null mice (*n* = 3 per group). Data are represented as the mean ± SEM. **P < 0.01, using Students *t* test. ****P < 0.0001 using 2-way ANOVA with multiple comparisons.

To investigate the atrial structure of *Scn1b*-null hearts, we assessed atrial size and probed for evidence of atrial fibrosis using echocardiography and picrosirius red staining, respectively. Echocardiography analysis of P16–17 mice showed that, while ventricular size was reduced in the null animals (Supplemental Figure 4B), atrial dimensions, as well as systolic and diastolic function, were similar between genotypes (Supplemental Figure 4, A and C–F). Consistent with previous work (7, 31), the body weights of P16–17 *Scn1b*-null mice were reduced compared with WT (Supplemental Figure 5). Picrosirius red staining of histological coronal sections of the whole heart showed significant collagen deposition in the null atria compared with WT, suggesting fibrosis (Figure 2, A and B). In contrast, no differences in ventricular picrosirius red staining were observed between genotypes (Supplemental Figure 6). qPCR experiments showed increased expression of selected genes linked to fibrosis in the null right atrium compared with WT, including *Col1a1* (encoding collagen type 1 α1), *Col1a2* (encoding collagen type 1 α2), *Postn* (encoding periostin), *CTGF* (encoding connective tissue growth factor), *Vim* (encoding vimentin), and *Acta2* (encoding α-smooth muscle actin) (Figure 2C). The expression levels of *Col1a1*, *Col1a2*, *Postn*, *Vim*, and *Acta2* appeared to be increased in *Scn1b*-null left atrium WT but these values did not reach statistical significance (Figure 2C).

### Scn1b-null mice have increased susceptibility to pacing-induced AF.

**Figure 3:**
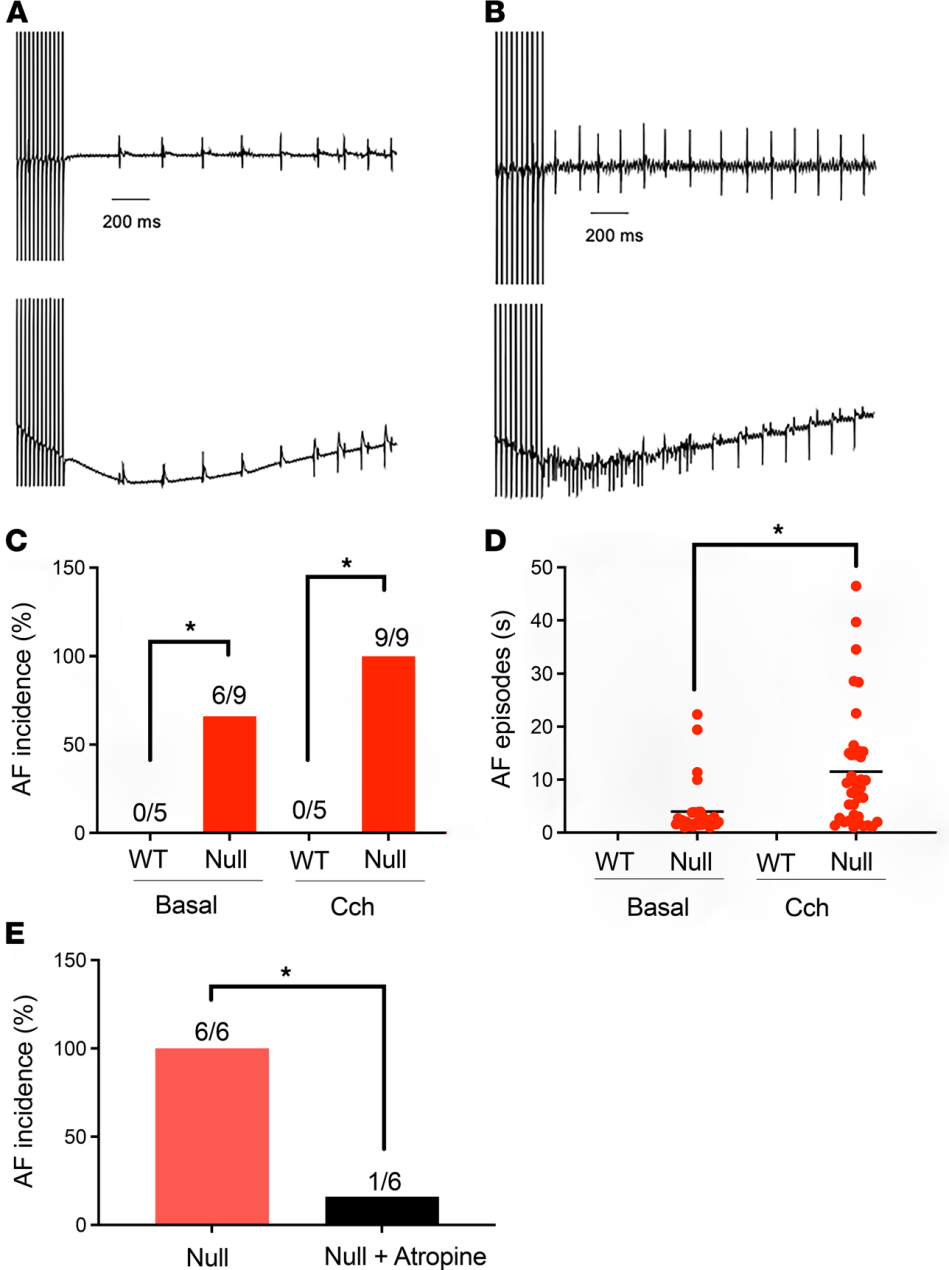
Neonatal *Scn1b*-null mice have increased susceptibility to AF in vivo. (**A**) Representative recordings of surface ECGs (top) and atrial electrograms (bottom) from P16 WT or *Scn1b-*null mice. Atrial burst pacing was delivered at 50 Hz for 2 seconds. A period of AF in the null mouse is indicated in red. (**B**) AF incidence under basal and vagotonic stimulation. AF testing was performed before and after i.p. administration of carbachol (Cch) (0.025 mg/kg). (**C**) Durations of basal and induced AF episodes in the mice shown in **B**. Each dot represents a single episode. Mean values are indicated by the bar. (**D**) Incidence of AF in the null mice before (red) and after i.p. administration of atropine (blue, 1 mg/kg). (**E**) Incidence of AF in the null mice before and after i.p. administration of atropine (1 mg/kg). Numbers of animals tested are shown in the bar graphs. *P < 0.05 using Fisher’s exact test.

We performed intracardiac recordings in P16 mice to test the hypothesis that fibrosis in neonatal null atria may provide an anatomical substrate for AF. We compared the incidence of AF between genotypes using atrial burst pacing (50 Hz for 2 s) before and after the i.p. administration of carbachol. The efficacy of carbachol was assessed in each mouse by the observation of decreased heart rate following drug administration. AF was defined as rapid atrial activity and high R-R interval variability. We observed AF in the ECG (Figure 3A) and atrial electrogram (Figure 3A), respectively, for null but not WT animals. Only AF episodes lasting more than 1 second were counted as such and an animal was considered to be susceptible to AF if at least 2 AF episodes were elicited during the experiment. Under basal conditions, we observed a significantly higher incidence of AF in the null mice (6 of 9) compared with WT (0 of 5) (Figure 3B). Following the administration of carbachol, AF was inducible in 100% of the null mice tested (9 of 9) compared with 0 of 5 WT, with prolongation of the AF episodes in null hearts under both conditions (Figure 3, C and D). Supplemental Figure 7 shows representative ECG and atrial cardiogram traces with AF in a null mouse in response to carbachol administration. In a separate experiment, we administered atropine (1 mg/kg) to null mice in which AF was found to be inducible under basal conditions. Atropine administration prevented subsequent pacing-induced AF episodes in 5 of 6 null mice tested, with 1 mouse still showing AF episodes (Figure 3E). These data suggest that, in addition to fibrosis, altered vagal tone may contribute to the high incidence of atrial arrhythmia in *Scn1b*-null animals.

### Scn1b-null mice exhibit sinoatrial node dysfunction.

**Figure 4:**
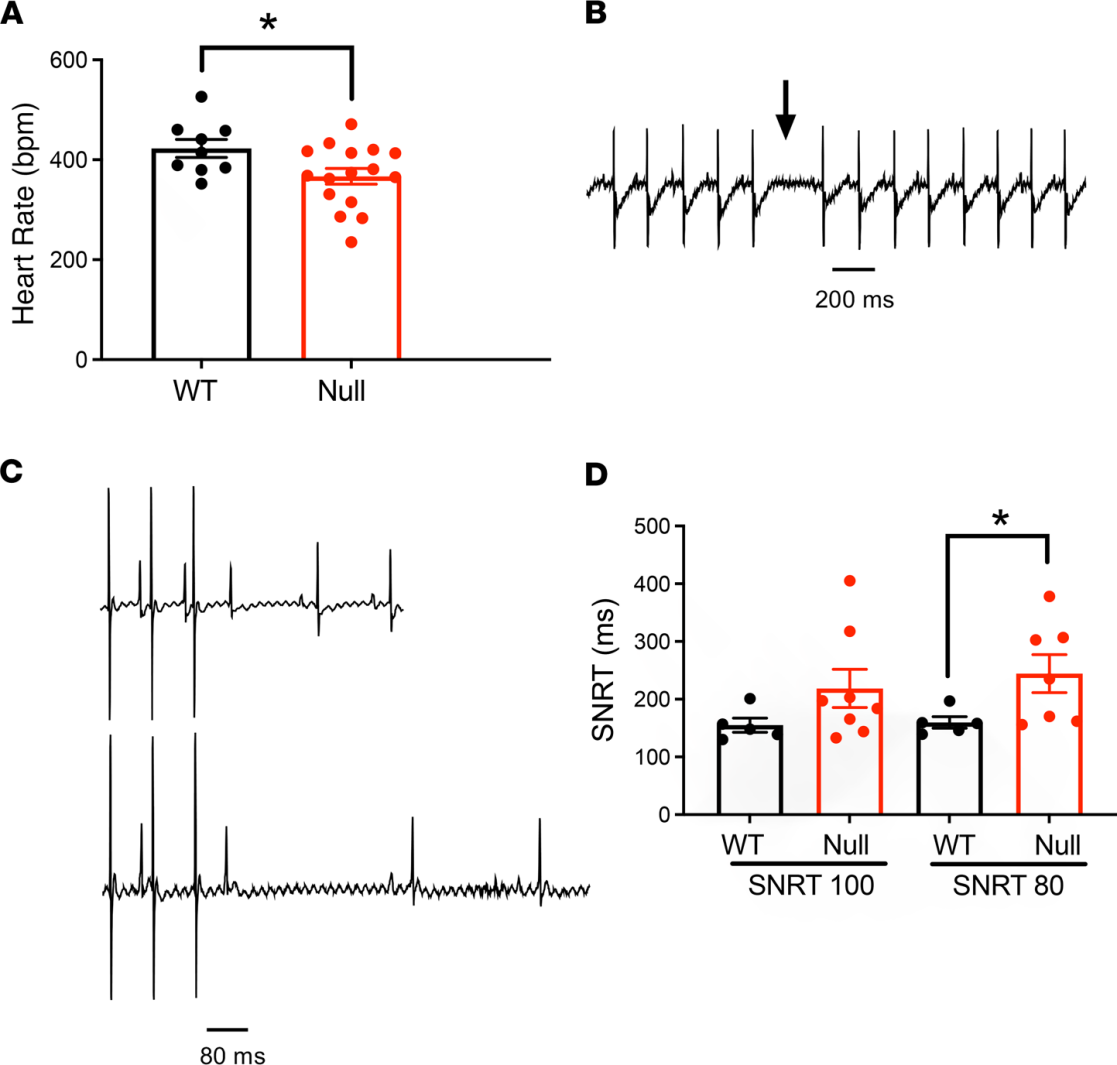
Neonatal *Scn1b*-null mice exhibit SAN dysregulation. (**A**) Comparison of heart rates between anesthetized WT (black) and null (red) mice at P16 (bpm). (**B**) Example of a sinus pause (arrow) during the monitoring of surface ECG in a null mouse. (**C**) Representative ECG recordings showing the time required to resume sinus rhythm following electrical stimulation, or SNRT. “P” indicates a P wave in each trace. (**D**) Comparisons of SNRT between genotypes assessed at cycle lengths of 100 or 80 ms. Each dot represents the value from 1 animal. Data are presented as mean ± SEM. *P < 0.05 using Student’s *t* test.

In a previous study comparing surface ECG recordings in anesthetized P17–18 *Scn1b*-null and WT mice, we reported that null mice had bradycardia, suggesting altered sinoatrial node (SAN) function, and prolonged corrected Q-T interval (QTc), suggesting altered ventricular function (7). Here, ECG analysis confirmed that P16 null mice had reduced HR (443.0 ± 18.0 WT vs. 367 ± 16 bpm null; *P* < 0.05; Figure 4A) and revealed the presence of sinus node pauses in 1 mouse (Figure 4B), both of which are hallmarks of SAN dysfunction. We conducted intracardiac recording to assess sinus node function by measuring sinus node recovery time (SNRT). As shown in the representative recording (Figure 4C), the time to resumption of sinus rhythm after electrical pacing was prolonged in *Scn1b*-null mice compared with WT. This difference in SNRT between genotypes was significant at the 80 ms pacing cycle length (160.0 ± 10 WT vs. 244.0 ± 33.0 ms null) (Figure 4D). Evaluation of ECG properties in animals at increasing postnatal developmental time points demonstrated that bradycardia and QTc prolongation become evident by P14 in the null mice (Supplemental Figure 8), suggesting that *Scn1b* deletion results in developmentally regulated alterations in SAN as well as ventricular function.

### Differences in Scn1b-null versus WT mouse cardiac neuroanatomy.

**Figure 5:**
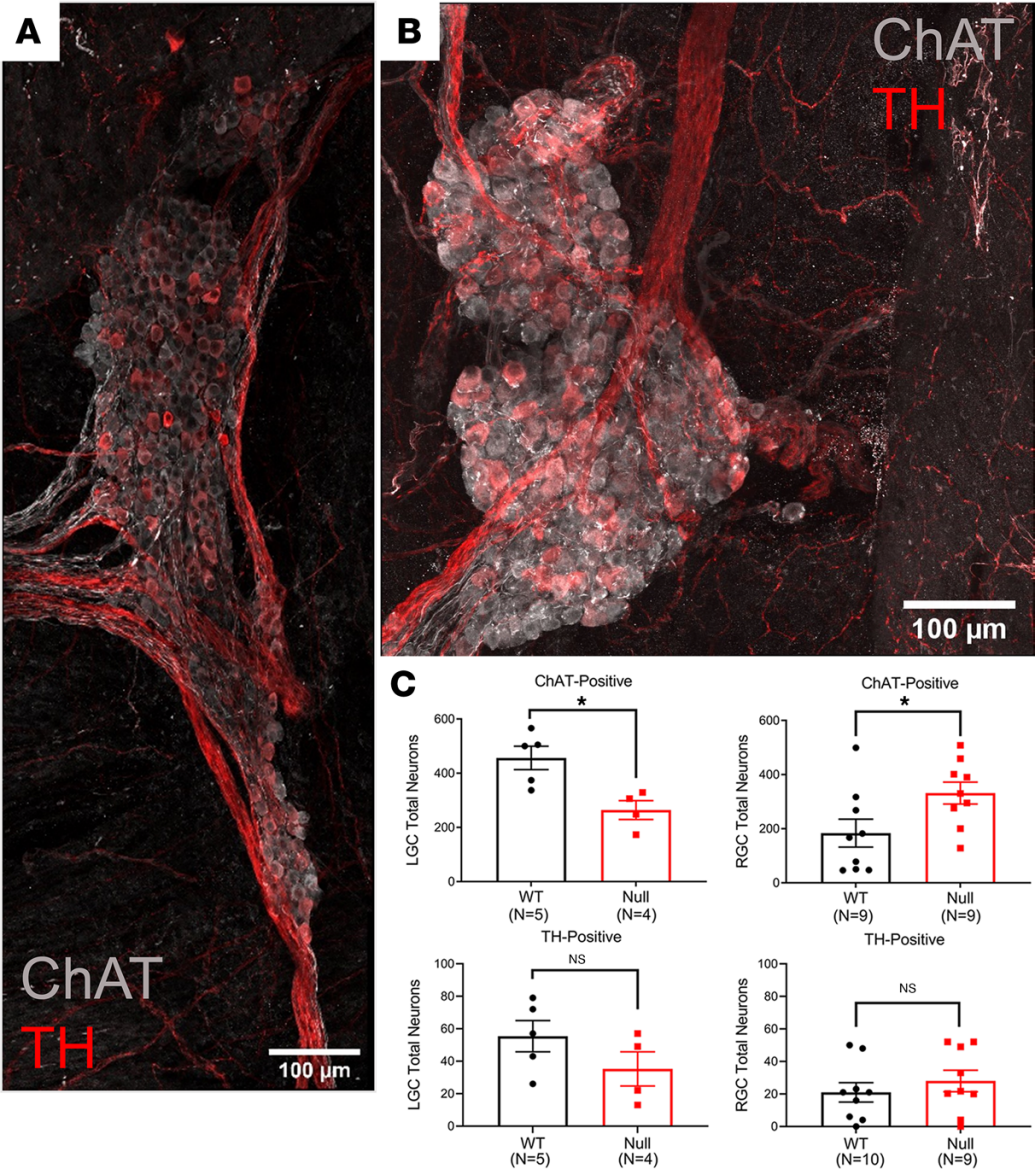
Immunofluorescence staining of murine atrial intrinsic ganglia. Merged, zoomed images from whole-mount atrial preparations shown in Supplemental Figure 9. (**A**) P16 *Scn1b-*null heart. (**B**) P16 WT heart. ChAT+ neurons labeled in gray; TH+ neurons labeled in red. Scale bars: 100 μm. (**C**) The mean and range of ChAT+ and biphenotypic intrinsic cardiac neurons are presented for all ganglia (LGC + RGC) versus the right (RGC) or left (LGC) ganglionic clusters from WT and null mouse whole mount atrial preparations. Data are presented as mean ± SEM. *P < 0.05 versus WT using Student’s *t* test.

Intrinsic cardiac nerves modulate the function of the cardiac conduction system, and autonomic nervous system dysfunction has been linked to several cardiac disorders (32–36). To investigate possible neuroanatomical differences in *Scn1b-*null atria compared with WT, we performed immunofluorescence confocal microscopy of whole-mount preparations of mouse atria stained with Abs to the sympathetic marker, tyrosine hydroxylase (TH), as well as the parasympathetic marker, choline acetlytransferase (ChAT) (Supplemental Figure 9). In agreement with previous work (37), we found cardiac neuronal soma to be either ChAT+, biphenotypic (ChAT+ and TH+), or TH+. Figure 5, A and B, show representative high magnification images of the right ganglionic clusters (RGCs), which are outlined in yellow in Supplemental Figure 9, for null and WT hearts, respectively. Representative merged images of ChAT staining (gray) and TH staining (red) are shown for each genotype, with separate ChAT (gray) and TH (red) images shown in Supplemental Figure 10. The total number of neurons (ChAT+ plus TH+ staining) in the left ganglionic clusters (LGCs) and RGCs combined (LGC + RGC) was comparable between genotypes (Figure 5C). However, we found genotypic differences in the number of ChAT+ neurons located in the LGC and RGC, respectively. We observed a significant increase in the number of ChAT+ neurons located in WT LGC compared with null mice (Figure 5C). In contrast, we observed a significant increase in the number of ChAT+ neurons located in the null RGC compared with WT (Figure 5C). We found no differences between genotypes in the number of neurons that were TH+ alone (Supplemental Table 1). Previous work has shown that the SAN is predominantly innervated by the RGC (38, 39), while the LGC predominantly innervates the atrial ventricular nodal (AVN) region (38). In Supplemental Figure 9, white arrowheads point to the intrinsic cardiac nerves from the RGC that extend toward the SAN. Taken together, our results suggest that increased numbers of ChAT+ neurons in the null RGC could alter SAN function and influence heart rate.

### Scn1b-null atrial myocytes show altered excitability.

**Figure 6:**
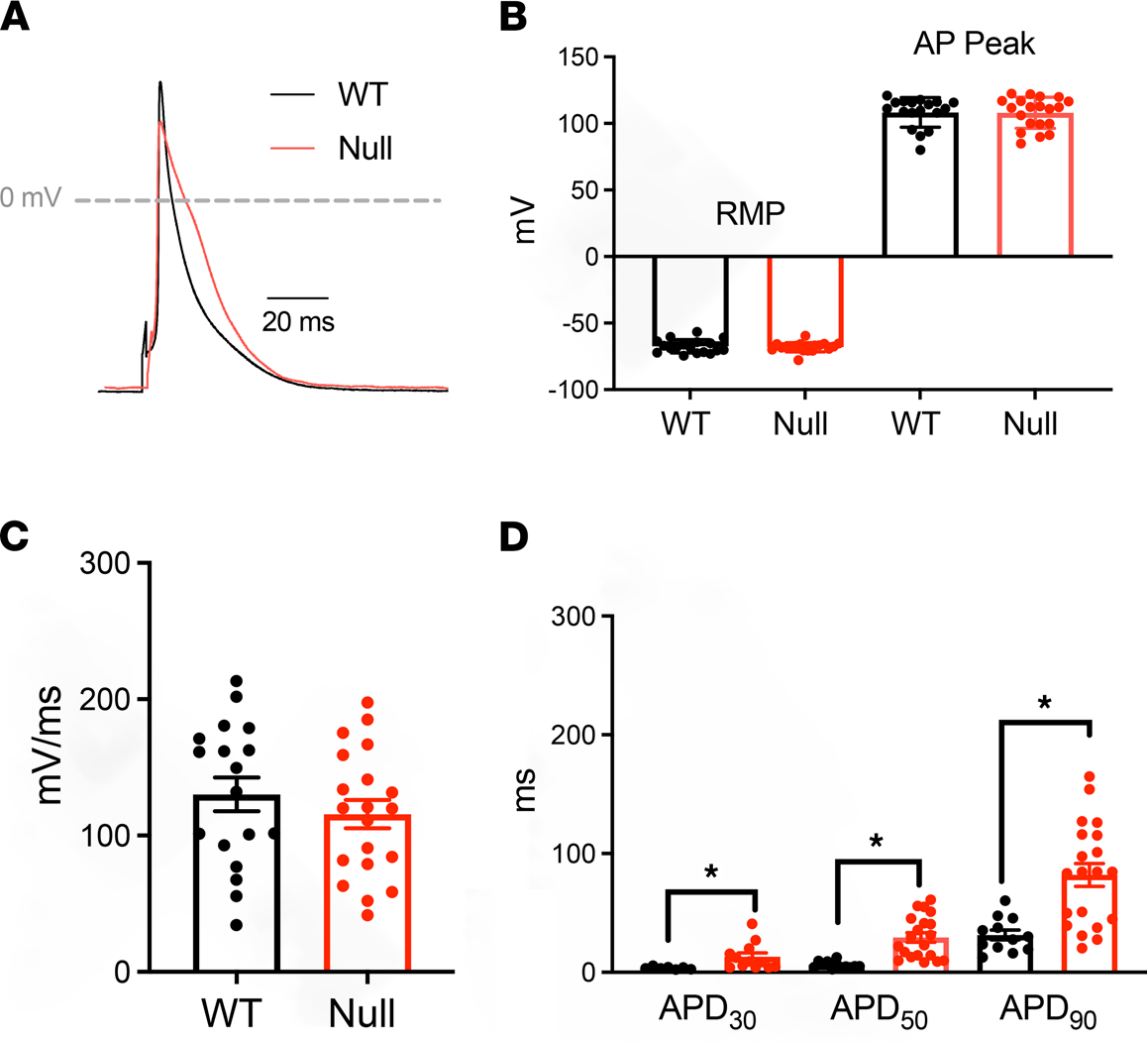
Neonatal *Scn1b-*null atrial myocytes show AP prolongation. (**A**) Representative recordings of APs from acutely isolated P15–18 WT (black) or *Scn1b*-null (red) atria myocytes. Scale bar: 30 ms. (**B**) Resting membrane potential (RMP) and AP amplitude values for WT and null atrial myocytes. (**C**) AP upstroke velocity (dV/dt) values for WT and null atrial myocytes. (**D**) APDs at 30%, 50%, and 90 % of membrane repolarization for WT and null atrial myocytes. Each dot represents the value from 1 cell. The number of myocytes (n) and mice (N) used are indicated in the inset. Data are presented as mean ± SEM. *P < 0.01 versus WT using Student’s *t* test.

**Figure 7:**
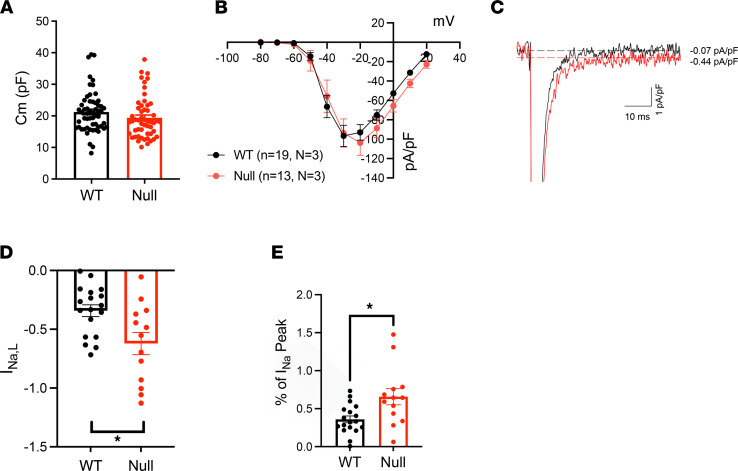
Neonatal *Scn1b-*null atrial myocytes have increased *I_Na,L_*. (**A**) Membrane capacitance (Cm) of P15–18 WT (black) or *Scn1b*-null (red) acutely isolated atrial myocytes. Plot shows combined Cm values obtained from the *I_Na_*, *I_Ca_*, and *I_K_* recordings. (**B**) I-V relationships for *I_Na,T_* are similar between genotypes. (**C**) Superimposed representative *I_Na,L_* recordings from a WT and null atrial myocyte. (**D**) Current densities for WT and null atrial myocyte *I_Na,L_* (*pA/pF*). (**E**) *I_Na,L_* expressed as percentage of *I_Na,T_*. Each dot represents the value from 1 cell. Data are presented as mean ± SEM. *P < 0.01 versus WT using Student’s *t* test.

We investigated the electrical properties of acutely isolated P15–18 null and WT mouse atrial myocytes. Action potentials (APs) were elicited at 1 Hz in current clamp mode. No differences in resting membrane potential, AP amplitude, or maximum upstroke velocity of the AP were observed between genotypes (Figure 6, B and C; and Table 1). However, null atrial myocytes showed significantly prolonged AP duration (APD) at 30% (APD30: 3.6 ± 0.4 ms WT vs. 13.0 ± 3.2 ms null; *P* < 0.05), 50% (APD50: 6.3 ± 0.8 ms WT vs. 29.4 ± 4.0 ms null; *P* < 0.05), and 90% (APD90: 31.4 ± 4.1 ms WT vs. 82.0 ± 9.6 ms null; *P* < 0.05) of membrane repolarization (Figure 6, A and D). No early afterdepolarization (EAD) waveforms were observed in either genotype. Voltage clamp experiments revealed no changes in atrial myocyte C_m_ between genotypes (Figure 7A). *I_Na,T_* density was similar between genotypes (Figure 7B and Table 2) but *I_Na,L_* density was significantly increased in the null myocytes (–0.36 ± 0.05 pA/pF WT vs. –0.66 ± 0.1 pA/pF null; *P* < 0.05) (Figure 7, C–E, and Table 2).

**Figure 8:**
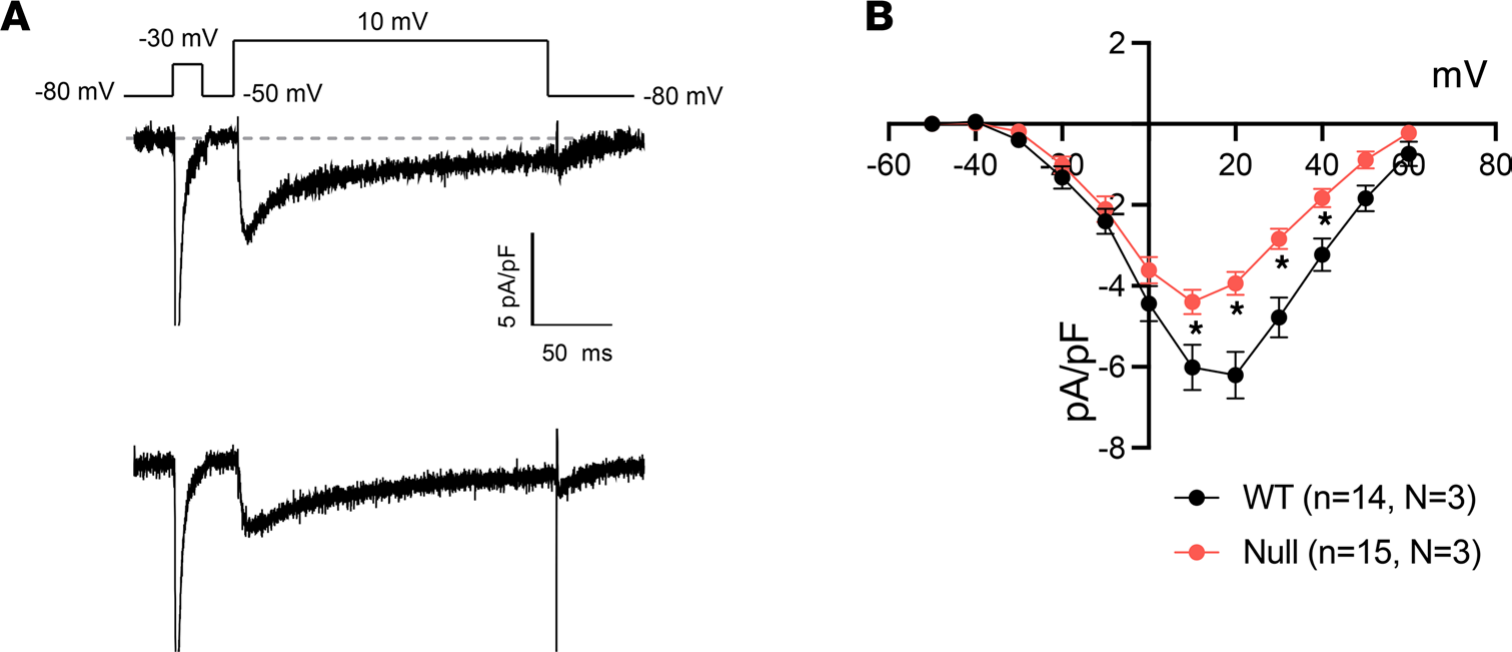
Neonatal *Scn1b-*null atrial myocytes have reduced *I_Ca,L_*. (**A**) Representative recordings of *I_Ca,L_* in P15–18 WT (top) or *Scn1b*-null (bottom) acutely isolated atrial myocytes. A short prepulse of –30 mV was delivered in the presence of 10 μM TTX to inactivate *I_Na_*. The membrane voltage was then set to –50 mV to ensure inactivation of *I_Na_* before delivery of voltage pulses to record *I_Ca,L_*. (**B**) I-V relationships for *I_Ca,L_* for WT (black) and *Scn1b*-null (red) atrial myocytes. The number of myocytes (*n*) and mice (*N*) used are indicated in the inset. Data are presented as mean ± SEM. *P < 0.01 versus WT using Student’s *t* test.

Differences in *I_Ca_* and *I_K_* can also contribute to AP prolongation. We investigated whether atrial *I_Ca,L_* was affected by *Scn1b* deletion. A –30 mV prepulse protocol plus the addition of 10 μM tetrodotoxin (TTX) to the bath solution were used to prevent current measurement contamination by *I_Na_*. Under these conditions, we found that acutely isolated null atrial myocytes had reduced *I_Ca,L_* density compared with WT (–6.0 ± 0.06 pA/pF WT vs. –4.4 ± 0.03 pA/pF null at 10 mV; *P* < 0.05; Figure 8, A and B). We also investigated possible differences in repolarizing *I_K_* between genotypes. We recorded transient and sustained outward *I_K_* from –30 to 60 mV, as well as inward *I_K_* that was sensitive to barium, from –120 mV to –30 mV. *I_TO_*, *I_KSUS_*, and *I_K1_* densities were similar between genotypes (Supplemental Figure 11).

## Discussion

*SCN1B* is expressed in both brain and heart in mice and in humans. LOF variants in *SCN1B* are linked to epilepsy and cardiac arrhythmia. *Scn1b*-null mice, which model DEE52, have severe seizures and early death plus a cardiac phenotype that includes bradycardia, QTc prolongation, increased *I_Na,T_* and *I_Na,L_* densities, altered *I_K_*, reduced *I_Ca_*, and altered calcium handling in acutely isolated ventricular myocytes (6–9). Importantly, cardiac specific *Scn1b*-null mice have a similar cardiac ventricular phenotype as the global null animals (8), demonstrating that arrhythmias are not secondary to seizures but rather that there are intrinsic differences in cardiac muscles resulting from *Scn1b* deletion. Taken together, this body of work has contributed to the hypothesis that both seizures and cardiac arrhythmias may contribute to SUDEP in patients with DEE52. The present study reports the first investigation of the effects of *Scn1b* deletion on cardiac atrial structure, function, and electrophysiology, modeling the effects of *SCN1B* LOF variants in the atrium. *Scn1b* deletion results in the differential expression of several genes that are associated with atrial dysfunction, leading to the prediction that *Scn1b* mice have abnormally formed atrial substrates that are conducive to arrhythmogenesis. We tested this hypothesis and found that *Scn1b-*null neonatal hearts have a significant accumulation of atrial collagen, indicating fibrosis and suggesting increased susceptibility to pacing-induced AF. We found that *Scn1b*-null neonates have SAN dysfunction and increased cholinergic innervation to the SAN compared with WT. Consistent with these findings, atropine administration prevented the reinduction of AF while carbachol administration prolonged AF duration in null animals. In agreement with previous work in atrial myocytes isolated from human patients with AF or from diabetic mice with AF (40, 41), we found increased *I_Na,L_* density, with no change in *I_Na,T_* density, in neonatal *Scn1b*-null atrial myocytes. We also found reduced *I_Ca,L_* and no change in *I_TO_*, *I_KSUS_*, or *I_K1_*. These data suggest that increased *I_Na,L_* may underlie the observed atrial APD prolongation in these cells. These important new results show that *Scn1b* plays a critical role in atrial as well as ventricular physiology early in life and suggest that *SCN1B* LOF variants may have devastating implications for the pediatric heart in addition to the developing brain (Graphical Abstract).

We observed increased *I_Na,L_* in acutely isolated neonatal *Scn1b*-null atrial myocytes compared with WT. While parallel translational studies have not been reported in pediatric patients, alterations in *I_Na,T_*, *I_Na,L_*, and VGSC α subunit expression have been reported in right atrial appendage myocytes isolated from adult patients with permanent AF (42). This work showed a 16% decrease in *I_Na,T_*, which was reflected in decreased expression of Na_v_1.5 protein. In addition, they reported a 26% increase in *I_Na,L_*, with a concomitant increase in Na_v_1.1 protein. It is not known whether changes in VGSC α subunit expression and function in adult patients with AF are causative or adaptive in response to high atrial excitability. However, our work suggests that increased *I_Na,L_* may contribute to the mechanism of atrial arrhythmia, at least for patients with biallelic *SCN1B* LOF. AF is the most common cardiac arrhythmia seen in clinical practice (23, 24). While AF occurs predominantly in the adult population, familial AF sporadically occurs in younger patients. There are reports of AF in patients with *SCN1B* variants, some of whom were diagnosed with AF before the age of 40 (43–45). A case report showed familial AF in a patient with double heterozygous variants in *SCN5A* and *KCNQ1*, respectively, at age 13 (46). Importantly, however, DEE52 is a pediatric disease that begins in the first year of life (4). Estimating that 1 human year is variably equivalent to 9 mouse postnatal days (47), the neonatal mice used in this study may be equivalent to 1- to 2-year-old children. Thus, this work is clinically relevant because it predicts that children with DEE52 may be at risk for developing atrial arrhythmias and should be evaluated periodically by a pediatric cardiologist. Our previous work using induced pluripotent stem cell cardiac myocytes derived from patients with DEE with variants in *SCN1A* predicted cardiac arrhythmia prior to clinical diagnosis in 1 of the patients (48). Taken together, our body of work supports the hypothesis that SUDEP in DEEs linked to VGSC gene variants is an arrhythmia of the brain and heart (49).

We were not able to induce AF in WT neonates, even with the administration of carbachol. Remarkably, neonatal *Scn1b*-null mice had a high incidence of pacing-induced AF, even in the absence of carbachol, and we were able to induce AF in all null mice tested following carbachol treatment. Atropine administration prevented pacing-induced AF episodes in 5 of 6 null mice. Bradycardia and SAN dysfunction have been linked to AF. Stimulating the vagus nerve to increase parasympathetic signaling can be both proarrhythmic and anti-arrhythmic (50). While low level vagus nerve stimulation, which does not induce bradycardia, can be anti-arrhythmic, parasympathetic over-stimulation that induces bradycardia can lead to proarrhythmic effects on the atrium (32, 51–53). These proarrhythmic effects include slowing of atrial conduction, shortening of the atrial effective refractive period, and increased dispersion of atrial refractoriness. This strategy is used to induce and maintain AF in experimental models (32). AF is often associated with sick sinus syndrome (54). Work in a dog model showed that persistent rapid atrial pacing to induce chronic AF resulted in SAN dysfunction, including prolongation of the SNRT and decreased heart rate (55). In human patients, prolonged SNRT after the termination of AF has been postulated to reflect the extent of atrial remodeling (56).

The intrinsic cardiac ganglia play important roles in triggering AF (57), and studies have shown that surgical ablation of these ganglia can terminate atrial arrhythmias (58–60). *Scn1b*-null mice have bradycardia that becomes evident by P14. Cardiac parasympathetic ganglia, glial cells, and nerve fibers in mice develop through the third postnatal week, the time point at which *Scn1b*-null mice die. Innervation of the AVN occurs earlier than the SAN, suggesting a critical role for early initiation of AVN conduction in the developing heart (61). Consistent with these results, P16 WT mice had increased numbers of ChAT+ neuronal cell bodies in the LGC, which predominantly innervates the AVN (38) compared with the RGC. We found the situation to be reversed in *Scn1b*-null atria, in which we observed a significant increase in the number of ChAT+ neurons located in the RGC, which predominantly innervates the SAN (38, 39) versus the LGC. We propose that neuronal migration and pathfinding may be impaired in the *Scn1b*-null atrium, and this will be the focus of future work. Our previous work using mouse cerebellar granule neurons showed that β1-β1 *trans* homophilic cell adhesion promotes neurite extension (22). *Scn1b*-null mice, which lack this signaling mechanism, have aberrant neuronal migration, pathfinding, and fasciculation in the cerebellum as well as in the corticospinal tract and hippocampus (62, 63). Thus, we suggest that differences in cardiac neuronal development, increased *I_Na,L_*, altered gene expression, and the development of fibrosis may synergistically contribute to AF susceptibility in *Scn1b*-null animals.

Our body of work shows that *Scn1b* and *Scn2b* are each necessary for normal atrial rhythm, however, there are important differences in the null mouse phenotypes. Adult *Scn2b*-null mice have atrial remodeling and increased susceptibility to AF (64). ECG measurements showed that adult *Scn2b*-null mice had normal heart rates compared with WT. In contrast, our present results show bradycardia in neonatal *Scn1b*-null mice. While AF induction in adult *Scn2b*-null mice required carbachol administration, we observed a high incidence of pacing-induced AF in neonatal *Scn1b*-null mice prior to the injection of carbachol. Similar to our observations in adult *Scn2b*-null mice, we found that the APD was significantly prolonged in neonatal *Scn1b*-null atrial myocytes with no effects on resting membrane potential or AP amplitude. We found that *I_Na,L_* was increased in *Scn1b*-null atrial myocytes, providing a possible mechanism for APD prolongation. In contrast, we found no differences in *I_Na_* density in *Scn2b*-null atrial myocytes compared with WT. Instead, downregulation of the noninactivating steady-state potassium currents, *I*_K_ and *I*_KSS_, was implicated in the delayed AP repolarization observed in this model. Finally, both *Scn1b* and *Scn2b* deletion appear to be linked to atrial fibrosis, suggesting that the ICDs of both β1 and β2 may regulate transcription in fibroblasts, although at different developmental time points.

In conclusion, we propose that biallelic *SCN1B* LOF variants that cause DEE may also increase susceptibility to atrial arrhythmias via a neurocardiac mechanism that includes aberrant cardiac innervation, SAN dysfunction, altered *I_Na,L_* leading to APD prolongation, and altered atrial structure through fibrosis. Combined loss of the multi-functional roles of the VGSC β1 and β1B subunits in channel modulation, cell-cell and cell-matrix adhesion, and transcriptional regulation in the heart as well as the brain, may help explain the complex mechanism of SUDEP. Further studies including using atrial-specific or SAN-specific *Scn1b*-null mice, which may have longer life spans and thus be suitable for more detailed physiological analyses, or knock-in mice expressing selected *SCN1B* DEE52 patient variants are necessary to explore whether targeting VGSC β1 regulation in the heart will open new therapeutic avenues to reduce SUDEP risk in patients with DEE52.

## Methods

### Animals

*Scn1b*-null and WT littermate mice were generated from *Scn1b^+/–^* mice that were congenic on the C57BL/6J background for over 20 N generations (6). Animals were housed in the Unit for Laboratory Animal Medicine at the University of Michigan Medical School. Male and female pups were used in all experiments.

### RNA-Seq

RNA was isolated from the atria of 4 P16 *Scn1b* WT and 4 P16 *Scn1b*-null mice using the Qiagen RNeasy Plus kit according to the manufacturer’s instructions. Sequencing was performed by the University of Michigan Advanced Genomics Core, with libraries constructed and subsequently subjected to 151 bp paired-end cycles on the NovaSeq-6000 platform (Illumina). Data quality was assessed using FastQC (version v0.11.8). Reads were mapped to the reference genome GRCm38 (ENSEMBL) using STAR (v2.7.3a) and assigned count estimates to genes with RSEM (v1.3.2). The University of Michigan Bioinformatics Core prefiltered the data to remove genes with 0 counts in all samples. Differential gene expression analysis was performed using DESeq2. Genes and transcripts were considered differentially expressed if they met the following 3 criteria: the test status is “OK”, the false discovery rate is less than or equal to 0.05, and a log fold change of expression has an absolute value of at least 0.5849625. GO-term enrichments were performed using iPathway Guide (Advaita).

### qPCR

Atria from P16 *Scn1b*-null and WT mice were individually homogenized, and total RNA was isolated from samples using the Qiagen RNeasy Fibrous Tissue Mini Kit according to the manufacturer’s instructions. Atrial tissue was homogenized with a Tissue-Tearor (BioSpec Products) followed by lysis through a sterile, 18-gauge hypodermic needle and sonication. RNA samples were run on a NanoDrop One Spectrophotometer (Thermo Fisher Scientific) to ensure adequate concentration and purity and then stored at –80°C. cDNA was generated from 1 μg of RNA using Reverse Transcriptase SuperScript III (RT SS III), random primers (Invitrogen), and deoxyribonucleoside triphosphates (dNTPs; Invitrogen). RNA, random primers, and dNTPs were incubated at 65°C for 5 minutes. Salt buffers, 0.1 M DTT, RNase Out, and RT SS III were added, and reactions were incubated at 25°C for 5 minutes, at 50°C for 60 minutes, and at 70°C for 15 minutes. cDNA was diluted 1:3 in RNase-free water. qPCR was performed using SYBR Green (Applied Biosystems) and gene-specific primers (Integrated DNA Technologies) on a QuantStudio 7 Flex Real-Time PCR System (Applied Biosystems). Gene-specific measurements of each cDNA sample were run in triplicate, along with the endogenous control gene (Gapdh) used for normalization, and then compared with WT expression levels. The relative expression levels of each gene were quantified using the comparative threshold (2^–ΔΔCt^) method of quantification. Data are presented as the fold change in gene expression ± SEM. Statistical significance (P < 0.05) between genotypes was determined using a 2-tailed Student’s *t* test.

### Picrosirius red staining

P16–17 WT and *Scn1b*-null mouse heart coronal sections were cut from paraffin-embedded blocks at 5 μm of thickness. Following deparaffinization and hydration with xylene and graded alcohols, the slides were treated with 0.2% Phosphomolybdic Acid (Rowley Biochemical, F-357-1) for 3 minutes, directly transferred to 0.1% Sirius Red saturated in picric acid (Rowley Biochemical, F-357-2) for 90 minutes, then again directly transferred to 0.01N Hydrochloric Acid for 3 minutes. Slides were dehydrated and cleared through graded alcohols and xylene and coverslipped with Micromount (Leica, 3801731) using a Leica CV5030 automatic coverslipper. Images were acquired with Keyence BZ-X810 All-in-one fluorescence microscope using the seamless stitching method scanning the Picrosirius red heart sections. The percentage of fibrosis was quantified with ImageJ (NIH) software. Data are presented as mean ± SEM.

### Echocardiography

This work was performed as a fee-for-service by the University of Michigan Small Animal Phenotyping Core with protocols approved by the Institutional Animal Care and Use Committee (IACUC). Induction of anesthesia was performed in an enclosed container filled with 5% isoflurane. After induction, the mouse was placed on a warming pad to maintain body temperature. A dose of 0.5%–1.5% isoflurane was supplied via a nose cone to maintain a surgical plane of anesthesia. The hair was removed from the upper abdominal and thoracic areas with depilatory cream. Eye lubricant was applied to prevent corneal damage during prolonged anesthesia. An ECG and respiration were monitored via noninvasive resting electrodes. Transthoracic echocardiography was performed in the supine or left lateral position. Then, 2-dimensional, M-mode, Doppler and tissue Doppler echocardiographic images were recorded using a Visual Sonics Vevo 2100 high resolution in vivo microimaging system with a MS 550D transducer with a center frequency of 40 MHz and a bandwidth of 22–55 MHz. We measured a left ventricular (LV) ejection fraction from the 2-dimensional long axis view. In addition, we measured systolic and diastolic dimensions and wall thickness by M-mode in the parasternal short axis view at the level of the papillary muscles. Fractional shortening and ejection fraction were calculated from the M-mode parasternal short axis view. Diastolic function was assessed by conventional pulsed-wave spectral Doppler analysis of mitral valve inflow patterns (early [E] and late [A] filling waves). Doppler tissue imaging (DTI) was used to measure the early (Ea) diastolic tissue velocities of the septal and lateral annuluses of the mitral valve in the apical 4-chamber view. Left atrial volumes were calculated by applying a prolate ellipse method, using apical 4-chamber and parasternal long axis views.

### Atrial myocyte isolation

Each experimental mouse was euthanized by decapitation, the heart was removed, and the blood was cleared using gentle perfusion with cold HBSS (Gibco) supplemented with 10 mM HEPES and 1 MgCl_2_. The heart was cannulated through the aorta and retrograde perfused on a Langendorff apparatus for 5 minutes at 37°C. HBSS buffer containing 50 μg/mL Liberase (Roche) was perfused into the heart for 10–12 minutes at 37°C. Right and left atria were removed from the heart and placed in a 3 cm petri dish containing fresh enzyme. Digestion of the tissue was continued by gentle stirring with a stir bar at 37°C for 5 minutes. Digested tissue was passed through a plastic pipette to fully disperse the myocytes. Atrial myocytes were transferred into HBSS (Thermo Fisher Scientific) containing 10% FBS (stopping solution) and centrifuged at 250 x *g* for 2 minutes. Cells were suspended in fresh stopping solution and subjected to calcium reintroduction until reaching a final concentration of 1.2 mM CaCl_2_.

### ECG and programmed electrical stimulation

In vivo studies were performed in anesthetized mice (induction period 5.0 vol. %, maintenance 2.0 % vol. isoflurane in 0.5 L/min 100% O_2_.) After the disappearance of reflexes, mice were placed onto a temperature-regulated operating table. Platinum electrodes were inserted subcutaneously in the limbs and connected to a custom-ECG amplifier for standard leads I and II. Standard ECG parameters, including HR, P wave duration and R-R, Q-R-S, P-R, and QTc intervals were analyzed once the heart rate reached steady state, which occurred after approximately 3 minutes. ECG data acquired during 30 seconds of heart beats were then averaged. Mitchell’s formula for QT correction in mice was used: QTc = QT/(RR/100)1/2 (65). A 1.1 Fr Octapolar stimulation-recording catheter (Scisense , EP catheter) was inserted through the jugular vein and advanced into the right atrium and ventricle. An S1S1 protocol at 2 times the threshold of capture was applied to determine the basal SNRT at 100 ms and 80 ms cycle lengths (SNRT_100_ and SNRT_80_). Atrial arrhythmia was assessed by atrial burst pacing for 2 seconds at 30, 40, or 50 Hz before and after the i.p. application of carbachol (0.025 mg/kg) or atropine (1 mg/kg). AF was defined as the occurrence of rapid and fragmented atrial electrograms (lack of regular P waves), with irregular AV-nodal conduction and ventricular rhythm, lasting at least 1 second.

### Whole cell patch clamp recordings

Patch pipettes with resistance between 2.0–2.5 MΩ were used for patch clamping experiments. Membrane capacitance (C_m_) was estimated by applying a 100 ms pulse from –100 to –120 mV before and after the rupture of the patch. Integration of the difference between these values was proportional to C_m_. After establishing the whole cell configuration, membrane capacitive components were canceled (~95%) by analog subtraction with the amplifier’s circuit (Axon multiclamp, 700B; Digidata, 1440), while the remaining linear components were subtracted with P/N protocol (Clamplex 10.7). Cardiomyocytes exhibiting both an estimated cell membrane resistance of greater than 700 MΩ and series resistance (Rs) of less than 5 MΩ were used for data acquisition. To attenuate the voltage error that results from the product of Rs and membrane current (*I_m_*), the Rs compensation (40%–50%) was applied through the amplifier. 

#### I_Na_ recording.

*I_Na_* was elicited by applying repetitive squared 200 ms pulses ranging from –70 to 60 mV from a holding potential of –120 mV at RT. The pipette solution contained (in mM): 120 CsCl-Asp, 1 NaCl, 10 EGTA, 1 MgCl_2_, 1 Na_2_ATP, 10 TEA-Cl, and 10 HEPES (pH 7.2 with CsOH). The bath solution contained (in mM): 15 NaCl, 50 CsCl, 20 TEA-Cl, 1.2 CaCl_2_, 60 Choline-Cl, 1 MgCl_2_, 1 4-aminopyridine, 0.05 CdCl_2,_ and 10 HEPES (pH 7.4 with CsOH). Nifedipine at a final concentration of 10 μM was added to the bath to block L-type calcium currents (*I_Ca,L_*).

#### I_Ca,L_ recording.

*I_Ca,L_* was recorded using repetitive squared 200 ms ranging from –50 to 60 mV preceded by a 30 ms prepulse of –30 mV to inactivate *I_Na_*. The pipette solution contained (in mM): 120 CsCl-Asp, 10 EGTA-Cs, 1 MgCl_2_, 1 Mg-ATP, 10 TEA-Cl, and 10 HEPES (pH 7.2 with CsOH) The bath solution contained (in mM): 137 NaCl, 5.4 CsCl, 1 MgCl_2_, 1.2 CaCl_2_, 10 HEPES, and 2 4-Aminopyridine (pH 7.4 with CsOH). 10 μM of TTX was added to the bath solution to ensure the block of *I_Na_*.

#### I_k_ recordings.

Transient outward potassium current (*I_to_*) was recorded using repetitive squared 300 pulses ranging from –40 to 60 mV for *I_to_*. Inward *I_K_* was recorded from –140 to –30 mV before and after the perfusion of BaCl_2_ (500 μM). *I_K1_* was defined as the *I_K_* that was sensitive to barium. The pipette solution for *I_K_* contained (in mM): 135 KCl, 5 K_2_-ATP, 10 EGTA-K, and 10 HEPES (pH = 7.2 with KOH). The bath solution contained (in mM): 5.3 KCl, 4.1 NaHCO_3_, 138 NaCl, and 100 CaCl_2_ (pH = 7.2 with KOH). 250 μM of CdCl_2_ and 10 μM of nifedipine were added to the bath solution to block *I_Ca,L_*.

#### AP recordings.

The threshold for AP initiation was determined by the application of 2 ms incremental current pulses from 100 to 800 pA. Steady AP capture was obtained by applying current pulses at 1.5x the threshold. APs were recorded at 1.0 Hz at RT. The pipette solution contained (in mM): 120 K-aspartate, 20 KCl, 1 MgCl_2_, 5 Na_2_ATP, 10 EGTA, and 10 HEPES (pH = 7.4 with NaOH). The bath solution consisted of 1x HBSS solution containing (in mM) 10 HEPES, 1 MgCl_2,_ and 1.2 mM CaCl_2_ (pH 7.4 with NaOH). Only myocytes with resting membrane potentials of at least –65 mV were included in the analysis.

### Whole-mount atrial preparations

Cardiac dissections were performed from euthanized mice as described previously (37, 38, 66) by an investigator blinded to genotype. In brief, the heart was perfused with cold PBS to ensure clearance of blood. The heart was then excised and placed in a Sylguard-coated dish containing cold PBS. The greater ascending vessels were separated from the atria. Then the atria were separated from the ventricles. The atria were gently dissected open, flattened, and fixed for 30 minutes in methanol at RT, and then rinsed 3 times for 10 minutes in cold PBS.

### IHC

Tissues were permeabilized for 1 hour in 0.5% Triton-X in PBS and blocked in 5% normal donkey serum in PBS for 2 hours at RT. The preparations were incubated in a mixture of primary antibodies (polyclonal goat anti–choline acetyltransferase [Millipore, anti-ChAT, 1:100] and polyclonal rabbit anti–tyrosine hydroxylase [Abcam, anti-TH; 1:750]) for 24–48 hours at 4°C. After washing, tissues were incubated in a mixture of secondary antibodies for 4 hours at RT. Tissues were then mounted on glass slides using Prolong Gold Mounting Medium (Invitrogen), coverslipped, and sealed with clear nail polish.

### Confocal microscopy and image analysis

Fluorescent images were acquired using a Nikon A1R inverted confocal microscope (Nikon Instruments) located in the University of Michigan Microscopy Core. Image analysis was performed using Fiji software. Neurons that had a clear soma were considered for analysis. The number of cells and identification of ChAT positive (+) or TH + signals were determined by manually counting cell bodies. Investigators were blinded to mouse genotype. The cell labeling of each cardiac ganglion was counted independently at least twice, and the results averaged.

### Statistics

Statistical analysis of picrosirius red staining was performed using Student’s *t* test. qPCR results were analyzed using Student’s *t* test. Statistical analyses of *I_Na_*, *I_Ca_*, *I_K_*, AP, and echocardiography data were performed using Student’s *t* test, assuming equal variances. ECG data were analyzed using 2-way ANOVA with Tukey’s post-hoc comparison test. Immunofluorescence data were analyzed with the Shapiro-Wilk test to test for normal distribution followed by 2-tailed Student’s *t* test. For all tests, a *P* value < 0.05 was considered to be significant.

### Study approval

All animal procedures were performed in accordance with NIH policy and approved by the University of Michigan’s IACUC. Investigators were blinded to genotype for all experiments.

### Data availability

RNA-Seq data have been submitted to the repository at NCBI GEO. Data will become publicly available upon acceptance of manuscript.

### Research materials availability

*Scn1b^+/–^* mice are available from the University of Michigan under the Materials Transfer Agreement.

### Author contributions

NE performed tissue isolation, whole mount preparation and imaging/analysis, and ECG recordings. RRM performed intracardiac recordings, ECG recordings, patch clamp electrophysiology, and analysis of echocardiography and provided mentoring support to NE. SH and CS performed RNA isolations and qPCR experiments and analyses. SW performed cardiac myocyte isolation, picrosirius red staining, imaging, and analysis, and qPCR and analysis. AAB performed the RNA isolation for RNA-Seq. CC performed picrosirius red staining and analysis. LJ and WRA contributed to whole mount image analysis. SN mentored and trained NE in whole mount preparation and imaging analysis. NRM and MSR provided mentorship and funding to RRM. LLI contributed to experimental design and interpretation and provided funding. LFLS provided mentoring and training to NE. NE, RRM, LFLS, and LLI cowrote the manuscript. NE and RRM are recognized as co-first authors. NE and RRM performed equal amounts of work and so their authorship is listed in alphabetical order.

## Supplementary Material

Supplemental data

## Figures and Tables

**Table 1:**
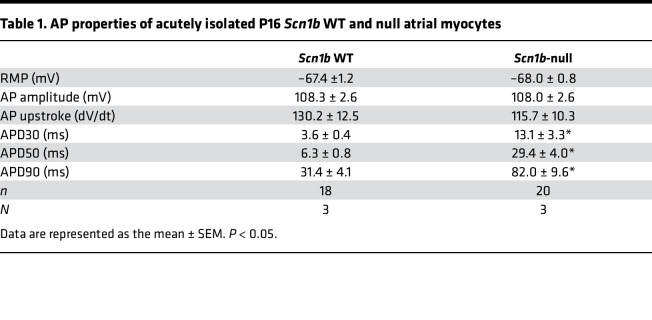
AP properties of acutely isolated P16 *Scn1b* WT and null atrial myocytes

**Table 2:**
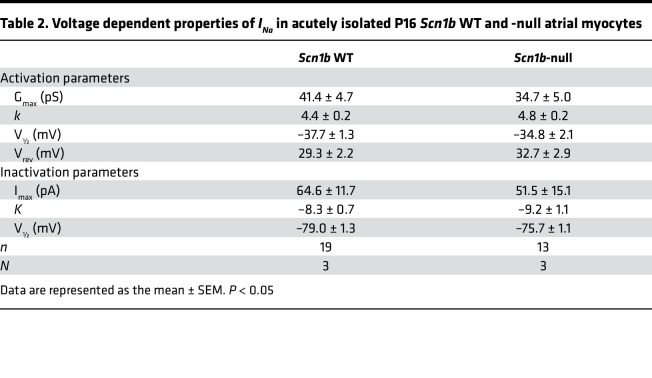
Voltage dependent properties of *I_Na_* in acutely isolated P16 *Scn1b* WT and -null atrial myocytes

## Version 1 05/23/2022

Electronic publication

## Version 2 03/11/2026

The following sentence was added to the Conflict of Interest: “MSR is a member of the Board of Directors at Eli Lilly.”
