# Supplementary material for: Neonatal Scn1b-null mice have sinoatrial node dysfunction, altered atrial structure, and atrial fibrillation
Source: JCI Insight. 2022 May 23;7(10):e152050. doi: 10.1172/jci.insight.152050 (PMC9220823; doi:10.1172/jci.insight.152050)
Supplement: Supplemental data [file jciinsight-7-152050-s011.pdf]

SUPPLEMENTAL MATERIALS

Supplemental Table

TH-Positive	**WT**			**Null**		
	**Mean ± SD**	**Range**	**n**	**Mean ± SD**	**Range**	**n**
LGC+RGC	80.60 ± 40.46	47.00 – 127.0	5	50.00 ± 32.59	27.00 – 98.00	4
RGC	21.00 ± 17.87	0.00 – 50.00	9	28.00 ± 19.81	0.00 – 52.00	9
LGC	55.40 ± 21.52	26.00 – 79.00	5	35.26 ± 21.08	13.00 – 57.00	4

Supplemental Table 1

The mean number and range of TH+ (TH only, with no ChAT staining) intrinsic cardiac neurons overall (LGC + RGC combined), in the RGC, or in the LGC from *Scn1b* WT or null mouse heart preparations. Data were analyzed with the Shapiro-Wilk test to test for normal distribution followed by Student's test. No significant differences were found between genotypes.

Supplemental Figures and Figure Legends

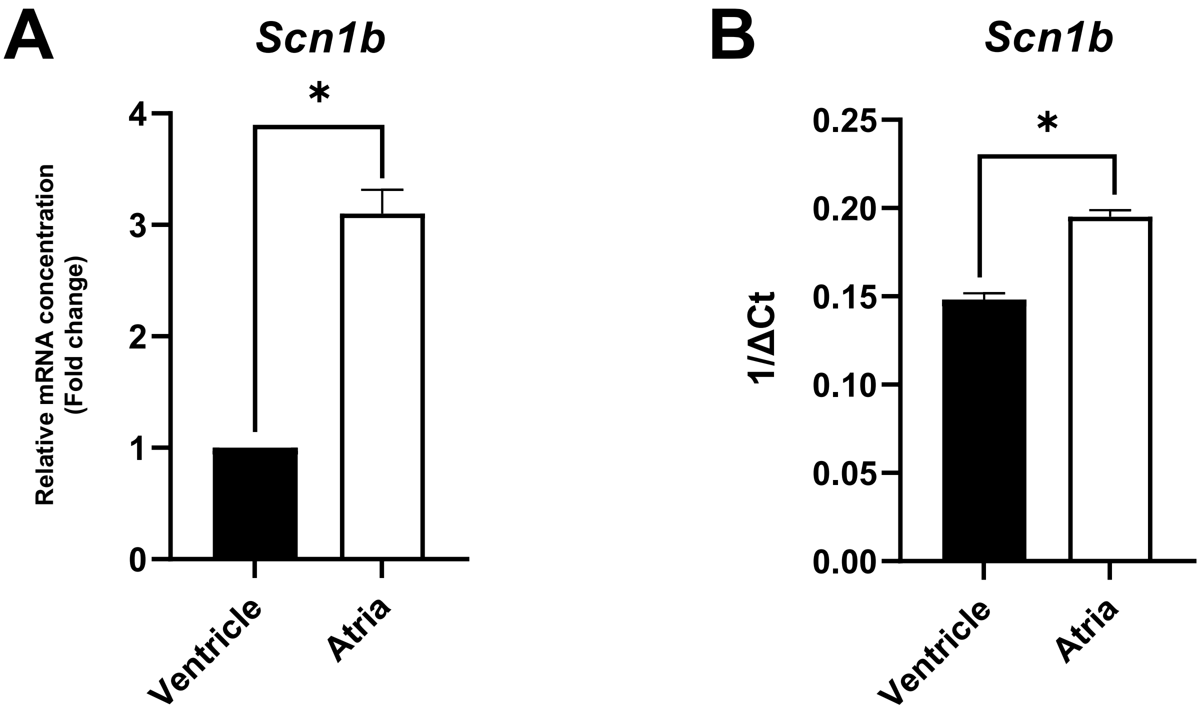

Supplemental Figure 1. *Scn1b* expression in P16 WT mouse atria vs. ventricles. A) Fold change in relative mRNA concentration. B) 1/ΔCt values. Data are represented as the mean ± SEM. Statistical significance was determined using Student's *t*-test (*p-value < 0.001).

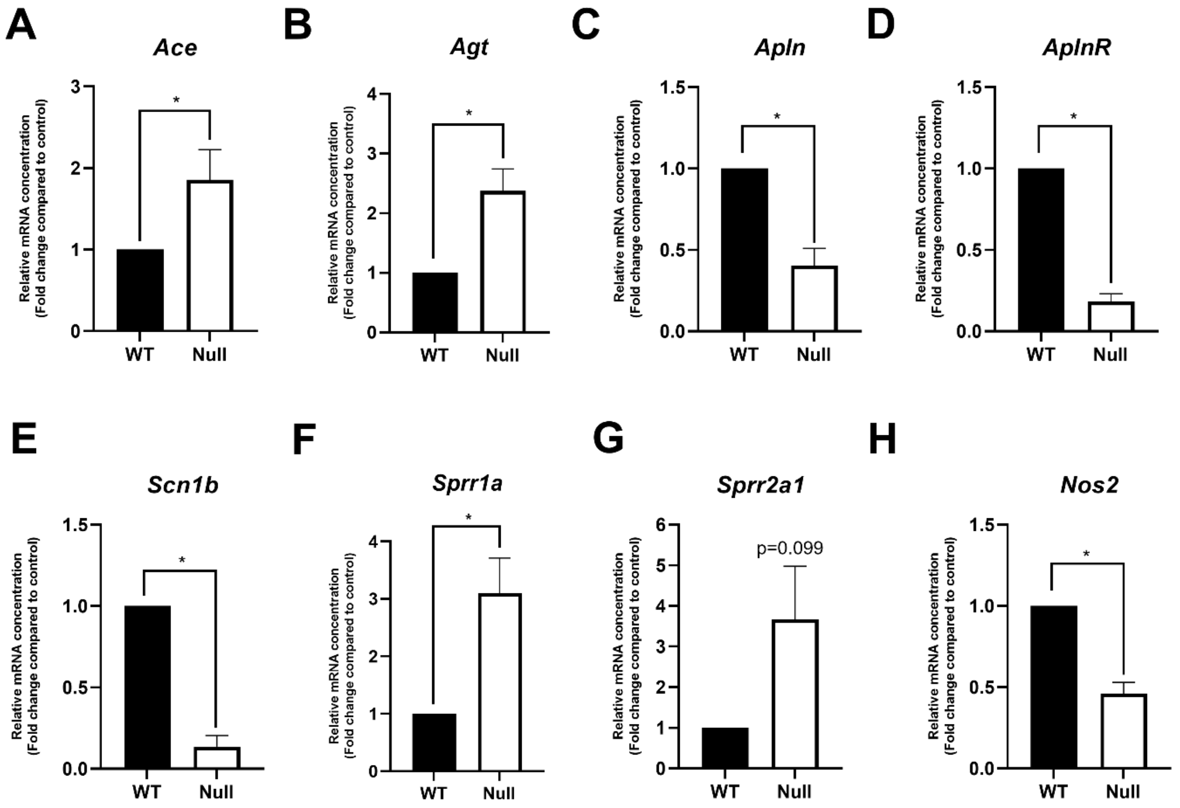

Supplemental Figure 2. **Differential expression of cardiac genes.** A-H) Confirmation of RNA-Seq data via RT-qPCR using 10 representative genes from P16 WT and *Scn1b* null atria. A) Relative expression of *Ace*. B) Relative expression of *Agt*. C) Relative expression of *Apln*. D) Relative expression of *AplnR*. E) Relative expression of *Scn1b* (confirmation of *Scn1b* deletion). F) Relative expression of *Sprr1a*. G) Relative expression of *Sprr2a1*. H) Relative expression of *Nos2*. Data are represented as the mean ± SEM. Statistical significance was determined using Student's *t*-test (*p-value < 0.05).

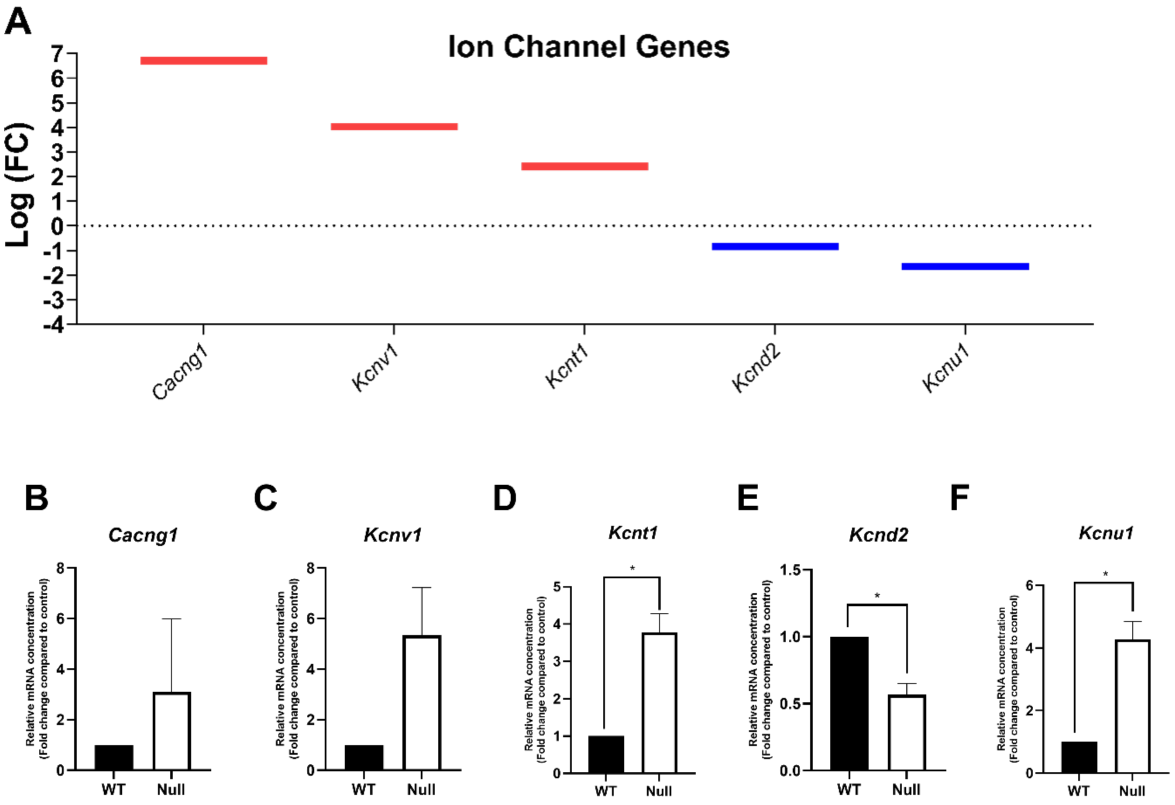

Supplemental Figure 3. **Differential expression of ion channel genes.** A) RNA-Seq data showing differential expression of ion channel genes in null atria compared to WT. B-F) RT-qPCR from P16 WT and *Scn1b* null atria. B) Relative expression of *Cacng1*. C) Relative expression of *Kcnv1*. D) Relative expression of *Kcnt1*. E) Relative expression of *Kcnd2*. F) Relative expression of *Kcnu1*. Data are represented as the mean ± SEM. Statistical significance was determined using Student's *t*-test (*p-value < 0.05).

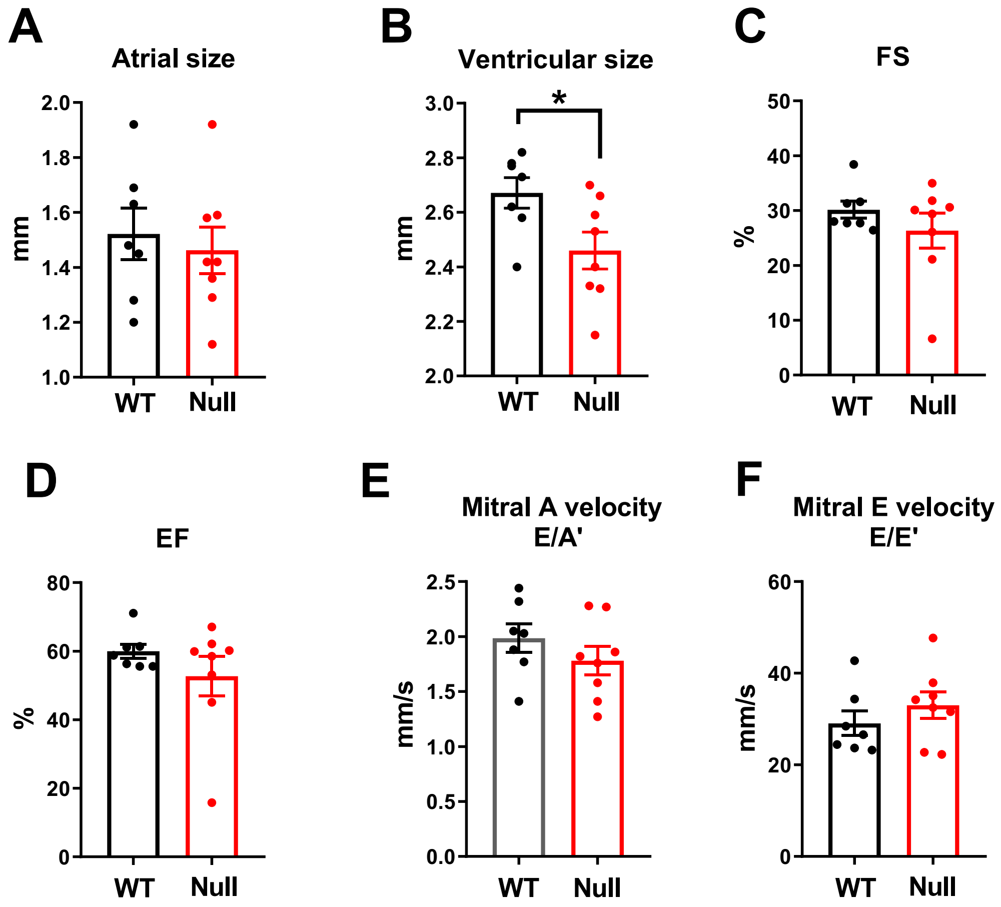

Supplemental Figure 4. Echocardiographic evaluation of P16-17 *Scn1b* null and WT mice. A-B) Atrial and ventricular dimensions calculated from two-dimensional echocardiography (2D-echo) for WT (black) and null (red) mice. C-D) Fractional release and ejection fraction determined by M-mode echocardiography. E-F) Early to late diastolic transmitral flow velocity (E/A) and E to early diastolic mitral annular tissue velocity (E/e') determined by Doppler echocardiography. Each dot represents the value from one mouse. Data are represented as the mean ± SEM. Ventricular size was reduced for null mice compared to WT. Statistical significance was determined using Student's *t*-test (*p-value < 0.05).

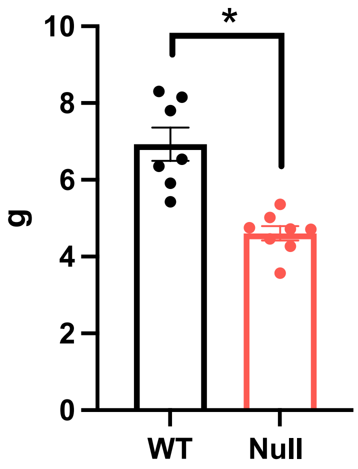

Supplemental Figure 5. Body weights of P16-17 WT and null mice. Data are represented as the mean ± SEM. Statistical significance was determined using Student's *t*-test (*p-value < 0.05).

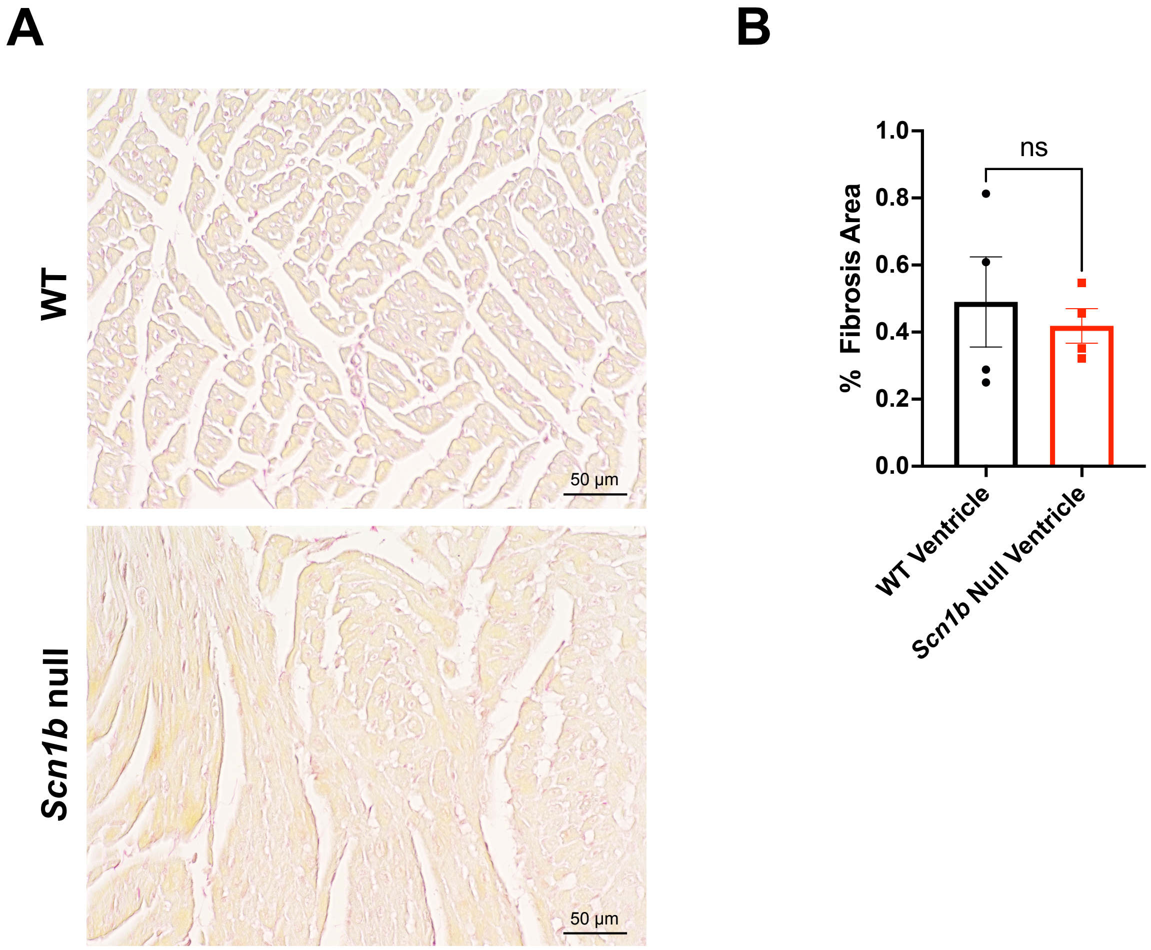

Supplemental Figure 6. Absence of ventricular fibrosis in P16-17 *Scn1b* null mice. A) Representative picrosirius stained longitudinal sections of ventricular tissue from WT and null mice. B) Quantification of collagen content in ventricles, presented as % Fibrosis area. Data are presented as mean ± SEM. Statistical analysis was performed using Student's *t*-test. Ventricular sections were generated from 3-5 mice per genotype. Each dot in the plot represents data from a single sample.

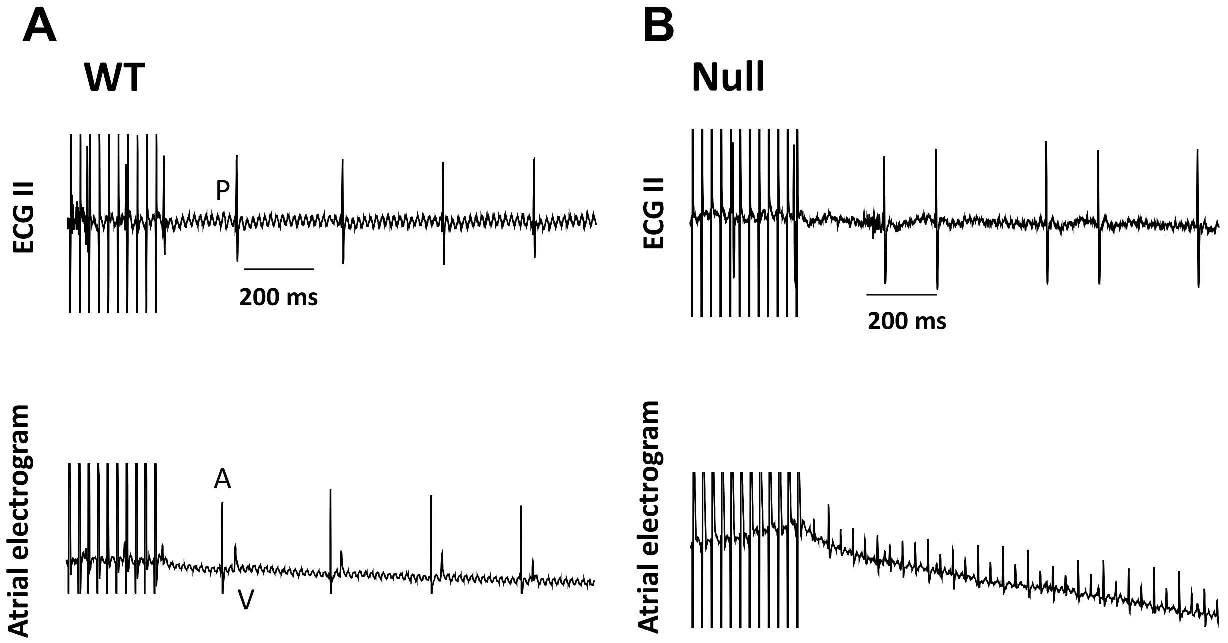

Supplemental Figure 7. Carbachol administration prolongs AF duration in *Scn1b* null mice. A) Representative ECG lead II (top) and atrial electrocardiograms (bottom) in P16 WT mice after i.p administration of carbachol (0.025 mg/kg). Sinus rhythm reassumed at the end of atrial pacing stimulation and no AF episodes were detected. B) Representative ECG lead II (top) and atrial electrocardiogram (bottom) in a P16 *Scn1b* null mouse after the i.p administration of carbachol (0.025 mg/kg). Electrical pacing in the presence of carbachol induced an episode of AF, characterized by the lack of evident P-waves in the lead II and high atrial activity and RR variability in the intracardiac electrogram. Scale bars: 200 ms.

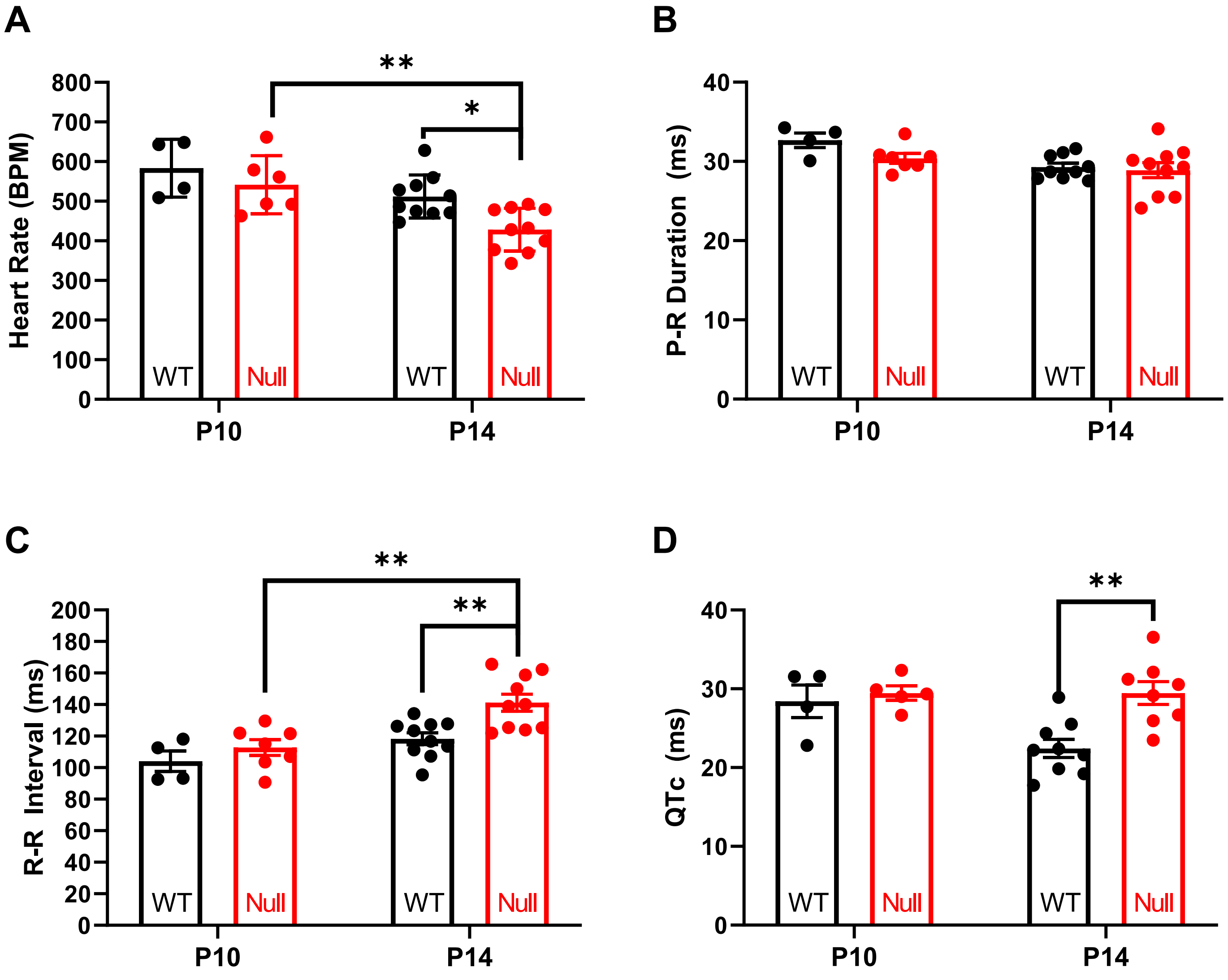

Supplemental Figure 8. Development of bradyarrhythmia and ventricular arrhythmia in *Scn1b* null mice. Surface ECG recordings from anesthetized P10 or P14 WT (black) or *Scn1b* null (red) mice. A) Heart rate, measured as beats per minute (BPM), decreased between P10 and P14 in the null mice. Heart rate was significantly different between genotypes at P14. B) P-R duration, measured in milliseconds (ms) was unchanged over time or between genotypes. C) R-R interval was prolonged in null mice at P14 but not at P10. D) QTc prolongation was observed in the null mice compared to WT at P14 but not at P10. Each dot represents one animal. Data are

presented as mean ± SEM. *$p<0.05$, **$p<0.01$ using two-way ANOVA with Tukey's post-hoc comparison test.

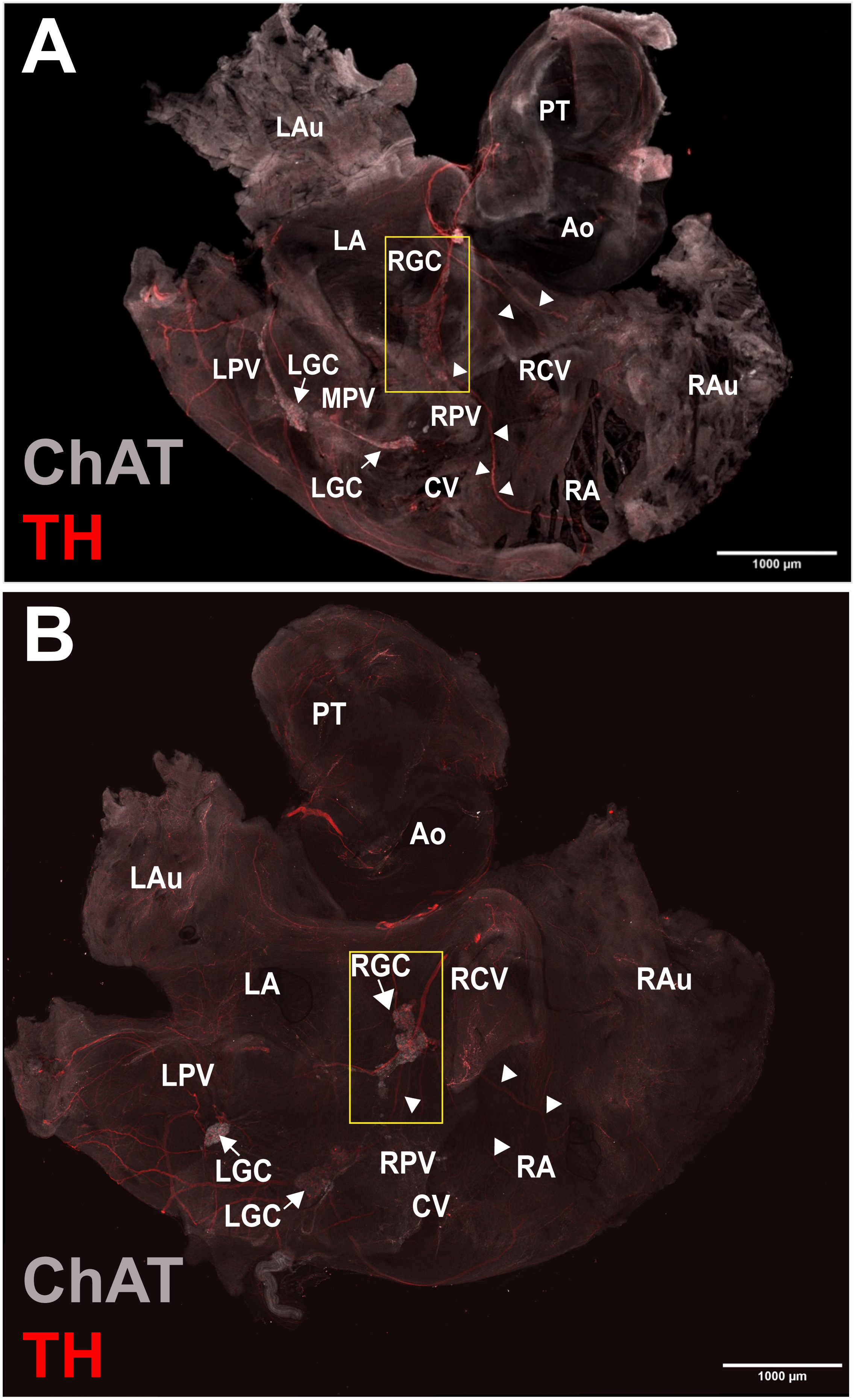
A
LAu
PT
LA
Ao
RGC
LPV
LGC
MPV
RCV
RAu
RPV
ChAT
LGC
CV
RA
TH
1000 μm
B
PT
Ao
LAu
LA
RGC
RCV
RAu
LPV
LGC
RPV
RA
LGC
CV
ChAT
TH
1000 μm

Supplemental Figure 9. Immunofluorescence staining of murine atrial intrinsic ganglia. Whole-mount atrial preparation from P16 *Scn1b* null (A) or WT (B) heart. ChAT+ neurons are labeled in gray; TH+ neurons are labeled in red. Arrows point to intrinsic ganglia; arrowheads point to nerves from the RGC that innervate the SAN. Scale bars = 1000 μm. Box in each panel shows the area that was examined at higher magnification in Figure 5 and Supplemental Figure X. Ao - ascending aorta; CV - caudal vein; LA - left atrium; LAu - left auricle; LCV - left cranial cava vein; LGC - left ganglion cluster; LPV - left pulmonary vein; MPV - middle pulmonary vein; PT - pulmonary trunk; RA - Right atrium; RAu - right auricle; RCV -right cranial cava vein; RGC - right ganglion cluster; RPV - right pulmonary vein.

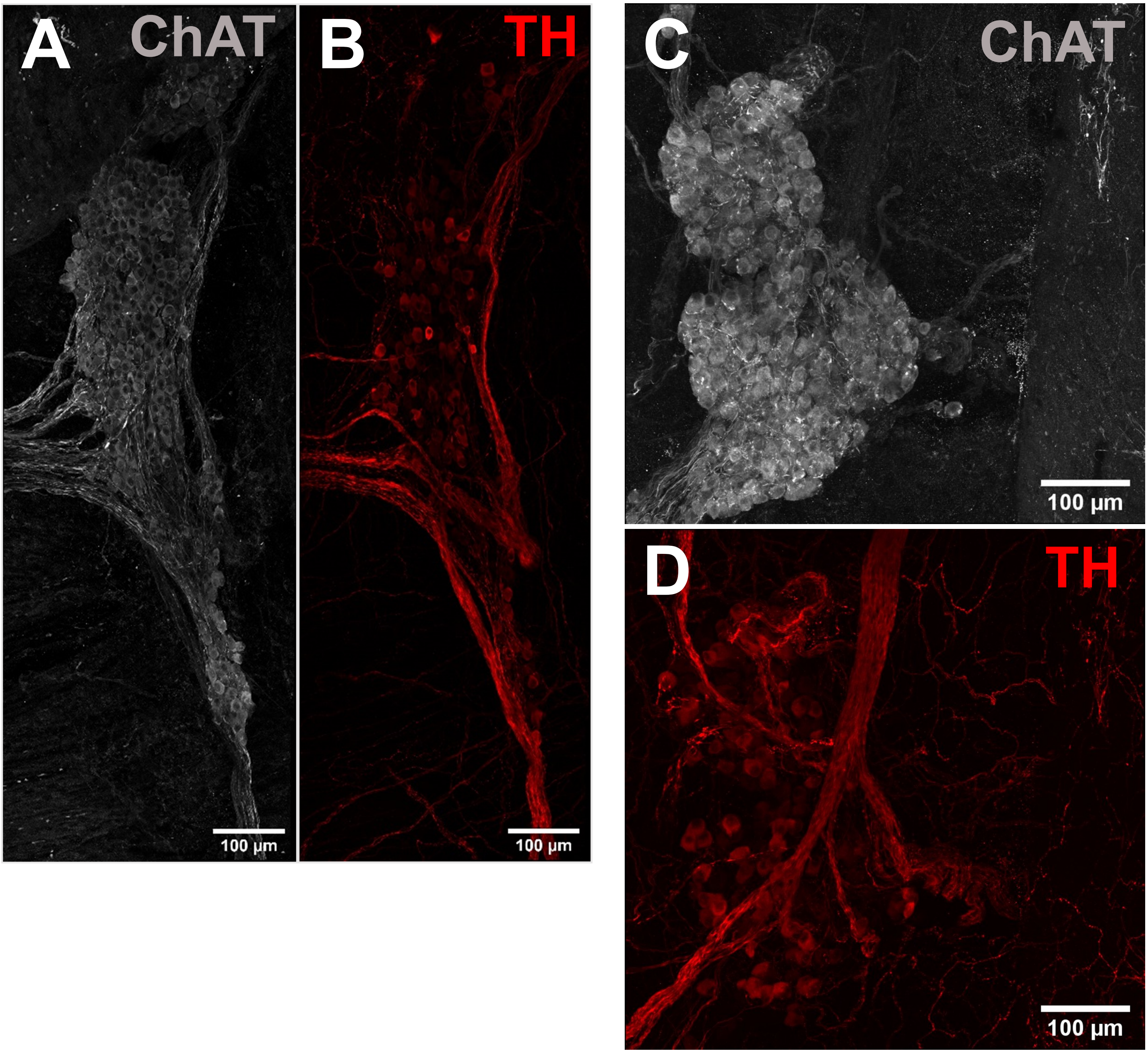

Supplemental Figure 10. Immunofluorescence staining of murine atrial intrinsic ganglia. Zoomed images from whole-mount atrial preparations indicated by boxes in Supplemental Figure 9. **(**A and B) P16 *Scn1b* null heart. (C and D) WT heart. A and C: ChAT+ neurons labeled in gray. B and D: TH+ neurons labeled in red. Scale bars = 100 µm.

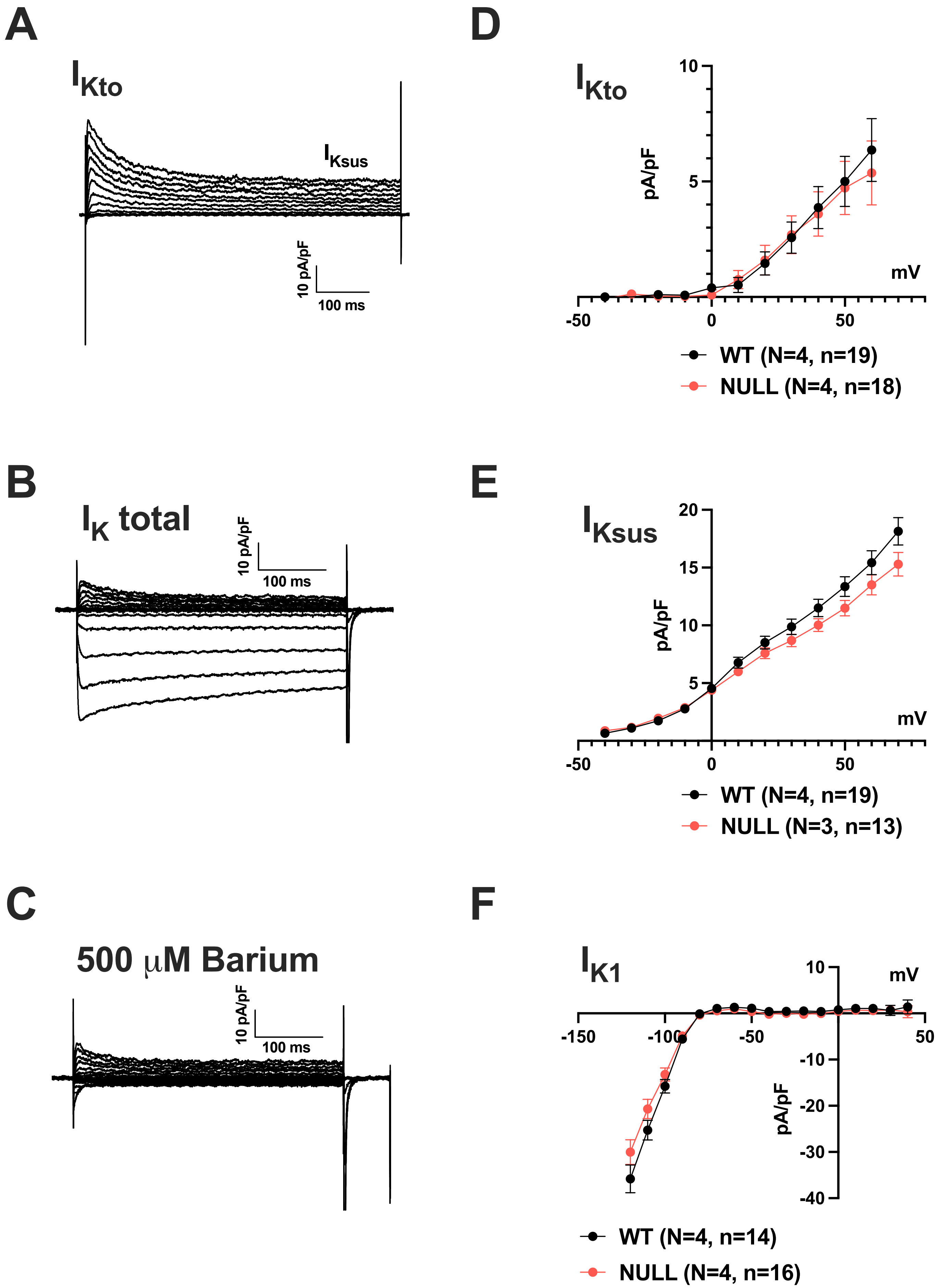

A
I_{Kto}
I_{Ksus}
10 pA/pF
100 ms
B
I_K total
10 pA/pF
100 ms
C
500 μM Barium
10 pA/pF
100 ms
D
I_{Kto}
pA/pF
mV
WT (N=4, n=19)
NULL (N=4, n=18)
E
I_{Ksus}
pA/pF
mV
WT (N=4, n=19)
NULL (N=3, n=13)
F
I_{K1}
mV
pA/pF
WT (N=4, n=14)
NULL (N=4, n=16)

Supplemental Figure 11. P15-18 acutely isolated *Scn1b* WT and null atrial myocytes have similar I_K. A) Transient and sustained families of I_K recorded in an acutely isolated WT atrial myocyte. B and C) Recording of inward and outward I_K before (B) and after (C) the perfusion of 500 µM $BaCl_2$. Barium-sensitive I_K was defined as I_{K1}. D-F) I-V relationships for I_{Kto} (D), I_{KSUS} (E), I_{K1} (F) for WT (black) and null (red) atrial myocytes. No significant differences were identified between genotypes. The numbers of myocytes (n) and mice (N) used are indicated in the insets. Data are shown as mean ± SEM.
